# Supplementary material for: Epigenetic modifications and metabolic gene mutations drive resistance evolution in response to stimulatory antibiotics
Source: Mol Syst Biol. 2025 Jan 16;21(3):294–314. doi: 10.1038/s44320-025-00087-4 (PMC11876630; doi:10.1038/s44320-025-00087-4)
Supplement: Supplementary file 1 — Appendix [file 44320_2025_87_MOESM1_ESM.pdf]

Appendix

Epigenetic Modifications and Metabolic Gene Mutations Drive Resistance Evolution in Response to Stimulatory Antibiotics

Hui Lin[a,b], *Donglin Wang*[a], *Qiaojuan Wang*[a,b], *Jie Mao*[a], *Lutong Yang*[a,b], *Yaohui Bai*[a,*], *Jiuhui Qu*[a]

[a] Research Center for Eco-Environmental Sciences, Chinese Academy of Sciences, Beijing 100085, China

[b] University of Chinese Academy of Sciences, Beijing 100049, China

[*] Corresponding author. E-mails: yhbai@rcees.ac.cn (Y. Bai)

Contents

This file contains Appendix Methods S1-S9, Appendix Figures S1-15 and Appendix Tables S1-12.

Appendix Method S1 Acquiring parameters of concentration-response curve (CRC) models .3
Appendix Method S2 Library Preparation and High-Throughput Sequencing.........................5
Appendix Method S3 Measurement of the Oxygen consumption rate (OCR) 7
Appendix Method S4 Estimation of population densities...8
Appendix Method S5 Growth fitness measurement by competition tests..................................9
Appendix Method S6 Biological transmission electron microscopy (TEM)............................10
Appendix Method S7 Resistance to heat and oxidative stress...11
Appendix Method S8 RNA sequencing (RNA-seq) analysis 12
Appendix Figure S1 13
Appendix Figure S2 14
Appendix Figure S3 15
Appendix Figure S4 16
Appendix Figure S5 17
Appendix Figure S6 18
Appendix Figure S7 19
Appendix Figure S8 20
Appendix Figure S9 21
Appendix Figure S10 22
Appendix Figure S11 23
Appendix Figure S12 24
Appendix Figure S13 25
Appendix Figure S14 26
Appendix Figure S15 27
Appendix Table S1 28
Appendix Table S2 29
Appendix Table S3 31
Appendix Table S4 35
Appendix Table S5 36
Appendix Table S6 37
Appendix Table S7 38
Appendix Table S8 40
Appendix Table S9 42
Appendix Table S10 43
Appendix Table S11 44
Appendix Table S12 46

Appendix Method S1 Acquiring parameters of concentration-response curve (CRC) models

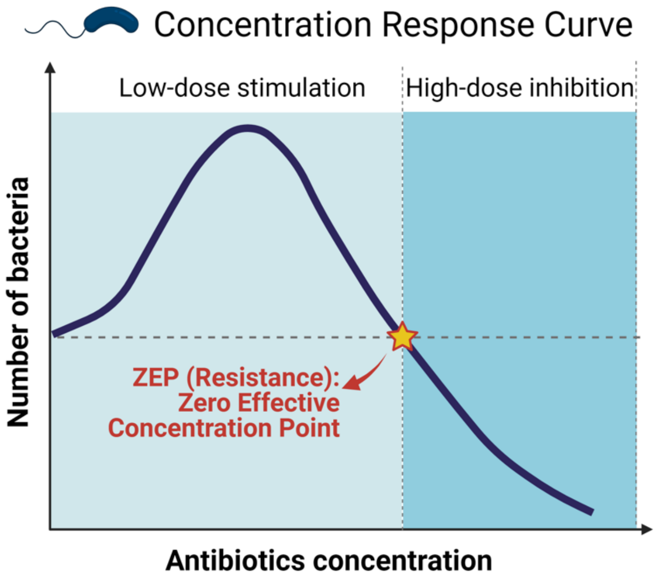

For a non-monotonic hormetic CRC, a seven-parameter function (Eq. (M2)), combining an ascending Hill function (Eq. (M1)) with a descending Hill function, was employed.

$$E(C) = E_m + \frac{E_{max} - E_m}{1 + \left(EC_{up}/C\right)^{H_{up}}} \tag{M1}$$

$$E(C) = \frac{\left(E_0 + \frac{E'_m - E_0}{1 + \left(\frac{EC_{down}}{C}\right)^{H_{down}}}\right) \times \left(E_m + \frac{E_{max} - E_m}{1 + \left(\frac{EC_{up}}{C}\right)^{Hup}}\right)}{E_m} \tag{M2}$$

where E_0, E'_m, EC_{down}, and H_{down} are the four parameters in the descending Hill function, depicting the minimum stimulatory effect, maximum stimulatory effect, median effective concentration that induces an effect of $(E_0 + E'_m)/2$, and Hill exponent, respectively. Given that often $E_0 = 0$ and $E_{max} = 1$, the equation reduces to a simpler form with fewer parameters: (Eq. (M3)).

$$E(C)=\frac{1}{1+\left(\frac{EC_{\text{down}}}{C}\right)^{H_{\text{down}}}}\times\left(E_m+\frac{1-E_m}{1+\left(\frac{EC_{\text{up}}}{C}\right)^{H_{\text{up}}}}\right) \quad \text{(M3)}$$

This model has been optimized using the Levenberg-Marquardt algorithm in MATLAB (R2018a). The optimization process involves fitting the model to empirical data to estimate the parameters such that the model accurately describes the hormetic effect observed (Wang, Liu et al., 2018).

Appendix Method S2 Library Preparation and High-Throughput Sequencing

(i) PacBio sequencing

For methylome analyses, evolved cells on days 15 and 25 were sent to Beijing Novogene Bioinformatics Technology Co., Ltd. (China) to obtain methylome data using single-molecule real-time (SMRT) sequencing (PacBio Core Enterprise). SMRTbell DNA template libraries were prepared following the Procedure & Checklist for 10-kb Template Preparation and Sequencing (Pacific Biosciences). In brief, the process involved DNA fragmentation and concentration, DNA damage and end repair, preparation of blunt ligation reactions, purification of SMRTbell Templates using 0.45X AMPure PB Beads, size selection via the BluePippin System, and post-size selection DNA damage repair. Library quality was assessed using a Qubit® 2.0 Fluorometer (Thermo Scientific) and insert fragment size was detected using an Agilent 2100 Bioanalyzer (Agilent Technologies). The prepared SMRTbell libraries were sequenced with a 120-min movie acquisition time, using a P4 polymerase-C2 DNA sequencing reagent kit following standard protocols for a PacBio RS II instrument (Pacific Biosciences). Genome-wide analyses for base modification and motif detection were performed using the default settings of the RS Modification and Motif Analysis.1 protocol included in SMRT Analysis v2.3.0 Patch 5. The FASTA reference genome sequence (*C. testosteroni* CNB-2; CP001220.2) used for base modification detection analyses was obtained from the NCBI database. The raw genome data were deposited in the NCBI GEO Short Read Archive (SRA) under

accession numbers PRJNA1104141.

(ii) Whole-genome sequencing

Whole-population sequencing was conducted to identify gene mutations in evolved cells on days 15, 35, and 55. Sequencing libraries were generated using a NEBNext® Ultra™ DNA Library Prep Kit for Illumina (NEB, USA) following the manufacturer's recommendations, and index codes were added to attribute sequences to each sample. FastQC (v0.11) was used to assess multiple quality control parameters, including read quality and GC content. Low-quality reads (< 20) were trimmed using Cutadapt (v2.10) (Martin, 2011). The reads were then mapped against the previously published reference genome using a Burrows-Wheeler aligner (BWA, v0.7.4)(Li & Durbin, 2009) with standard parameters. The alignments were then sorted into genomic positions and indexed using SAMtools (v1.3.1). Single nucleotide polymorphisms (SNPs) and insertions/deletions (InDels) were obtained by Breseq (v0.30.0) (Deatherage & Barrick, 2014). Genomic structural variants (SVs) were detected using BreakDancer and Varscan (v2.4.0)(Koboldt, Chen et al., 2009). The annotation files for *C. testosteroni* CNB-2 (GenBank: CP001220.2) were accessed from NCBI and were used to annotate the identified polymorphisms. The raw genome data were deposited in the NCBI GEO Short Read Archive (SRA) under accession numbers PRJNA1103939.

Appendix Method S3 Measurement of the Oxygen consumption rate (OCR)

OCR was quantified using an XFe96 Extracellular Flux Analyzer (Seahorse Bioscience). Overnight cultures of evolved isolates (G550) were first diluted in fresh NB media under SMX-free conditions until reaching an OD_{600} of approximately 0.1. These cells were then further diluted to an OD_{600} = 0.001, and 100 μL of the diluted cells were seeded onto XF cell culture microplates that had been precoated with 15 μL of poly-D-lysine (PDL) (Sigma). Following the addition of 100 μL of fresh media to each well, cellular respiration was quantified. OCR measurements were conducted at 6-minute intervals, alternating between 3 minutes of measurement and 3 minutes of mixing. NAD^{+}/NADH levels and byproducts of overflow metabolism were measured using liquid chromatography-tandem mass spectrometry as described previously.

Appendix Method S4 Estimation of population densities

To quantify colony-forming units (CFU) of *Comamonas testosteroni*, nutrient broth (NB)-agar plates supplemented with either sulfamethoxazole (sulfamethoxazole, 10 µg mL^{-1}, Sigma cat# S7507) or gentamycin (30 µg mL^{-1}, Sigma cat# E003632) were used to select for the *C. testosteroni* strains KF-1 and CNB-2, respectively. To identifying *Pseudomonas aeruginosa* colonies, *Pseudomonas* CN-agar plates (Hopebio cat# HB8484-2) were used, with colonies identified based on their distinct green color. CFU counting was conducted using traditional serial dilution and plating with glass beads on agar plates. Phosphate-buffered saline (PBS)-serial diluted cultures (10 µL, dilution factor ~10^5 to ~10^9) were plated on 90-mm agar plates with or without antibiotic selection.

Appendix Method S5 Growth fitness measurement by competition tests

Competition assays were carried out to assess interactions between pairs of metabolic CNB-2 mutants (G150, G350, and G550) and wild-type CNB-2, as well as the same set of mutants and kin bacterium KF-1 and *P. aeruginosa* PAO1, under both SMX-stressed and unstressed conditions. These metabolic mutants, characterized by confirmed genotypes, were derived from evolved populations. Competing strains were mixed at approximately equal ratios (1:1) and cultured in MSM medium at 30 °C with shaking at 170 rpm in darkness. After 24 h of co-cultivation, the mutant proportion was determined by counting colony-forming units (CFU) on selective media.

Appendix Method S6 Biological transmission electron microscopy (TEM)

Cell samples of G250 isolates developed under different evolution protocols and metabolically evolved mutants were immediately fixed after excision in 2.5% glutaraldehyde and stored at 4 °C for 12–24 h for primary fixation. Post-fixation, samples were immersed in a solution of 1% osmium tetroxide and 2% potassium ferrocyanide for 1–2 h, followed by thorough rinsing with 18.25 MΩ deionized water four times (15 min each). The dehydration process involved a series of acetone solutions, progressing from 30% to 100% in steps of 20%, with each step lasting 10 min and the final 100% acetone step repeated twice (20 min each). Embedding was conducted in stages, starting with a mixture of acetone and embedding medium at ratios of 3:1 and 1:1 at 37 °C for 1 and 3 h, respectively, followed by immersion in pure embedding medium at 37 °C overnight. The embedding molds filled with the medium and samples were polymerized in a 60 °C oven for 48 h. Ultrathin sections (70–90 nm) were then cut using a Leica UC7 ultramicrotome (Leica Microsystems, Germany), floated onto copper grids, and stained with uranyl acetate for 15 min and lead citrate for 10 min. The stained sections were examined under a Hitachi HT7800 TEM (Hitachi High-Technologies Corporation, Japan) for ultrastructural analysis.

Appendix Method S7 Resistance to heat and oxidative stress

Specific assays were used to investigate the resilience of metabolically evolved mutants to heat and oxidative stress. For the heat stress assays, 1 mL of overnight mutant cultures were incubated in a water bath at 40 °C to 70 °C for 30 min. For the oxidative stress assays, the cultures were exposed to hydrogen peroxide (H_2O_2) at concentrations of 5 mM and 35 mM for a similar duration. Following oxidative stress treatment, the cultures were diluted in NB medium enriched with 10 U/mL bovine liver catalase to effectively neutralize the remaining H_2O_2. The cell survival rate under these stress conditions was assessed by determining the number of CFUs before and after stress. This involved plating serial dilutions of the cell cultures on NB agar plates, with the CFU counting conducted according to the detailed plate counting method described above.

Appendix Method S8 RNA sequencing (RNA-seq) analysis

RNA-seq analysis was employed to measure transcriptional levels in *C. testosteroni* ancestrally and dynamically evolved isolates G150 and G250. To investigate the molecular basis of resistance linked to gluconeogenic metabolism, RNA-seq was also performed on wild-type cells grown on succinate versus gluconate in the presence of 1 mg/L SMX. To ensure uniformity in growth stages across samples, cells were collected at the logarithmic growth phase after 24 h. All strains were cultured overnight in three replicates. Total RNA extraction was performed using a Qiagen mini-RNA prep kit according to the provided instructions and stored at -80 °C prior to cDNA library construction. RNA concentration, integrity (RIN), and quality (RQN) were assessed with an Agilent 2100 Bioanalyzer. Only samples with RIN/RQN values exceeding 8.0 were sequenced using the Illumina HiSeq 2500 platform, generating 150 nucleotide paired-end reads. Sequencing reads were assembled and analyzed against the reference genome (GenBank: CP001220.2) using the NCBI Prokaryotic Genome Annotation Pipeline. Normalization was conducted by calculating fragments per kilobase million (FPKM), with significant gene expression changes identified at an FPKM fold-change ≥ 1 and a false discovery rate (FDR) q-value < 0.01. Gene set enrichment analysis software (v4.0.1) (JAVA version) was obtained from the Gene Set Enrichment Analysis website (http://software.broadinstitute.org/gsea/downloads.jsp). The expression dataset, phenotype class, and reference gene sets were loaded into the GSEA software. Gene sets with (FDR) q-values < 0.01 were considered significantly enriched.

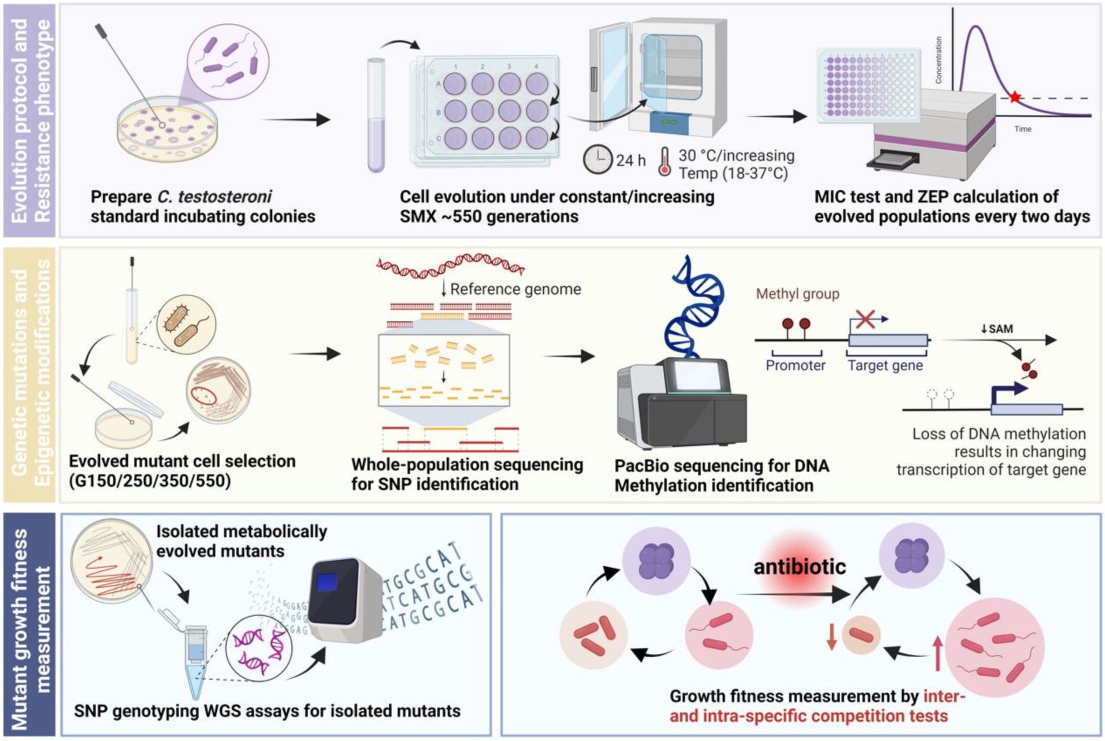

Appendix Figure S1. Experimental design.

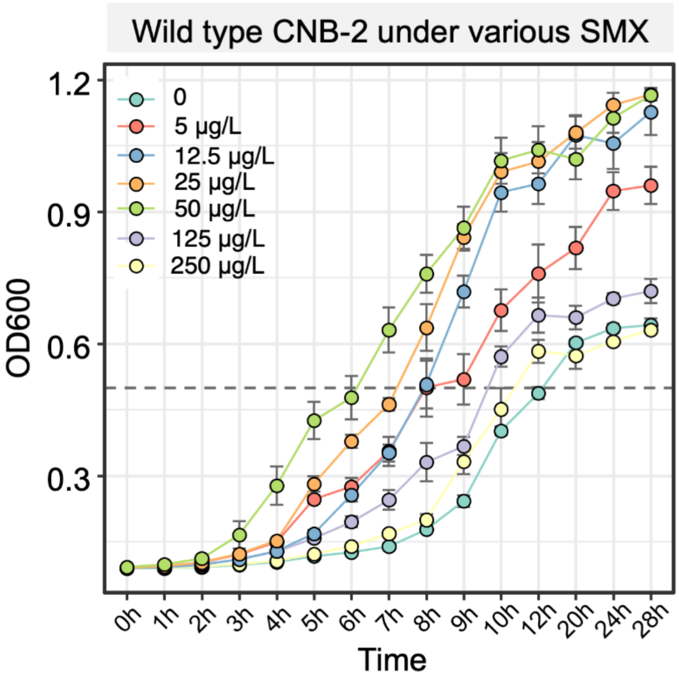

Appendix Figure S2. Growth curve of wild-type *Comamonas testosteroni* cultured in media containing sulfamethoxazole (SMX) concentrations below the Zero Effective Concentration Point (ZEP) of 250 μg·L^{-1}. Time series for the optical density (OD_{600}) of each species in monoculture is shown. Each evolutionary scheme comprised six biological replicates.

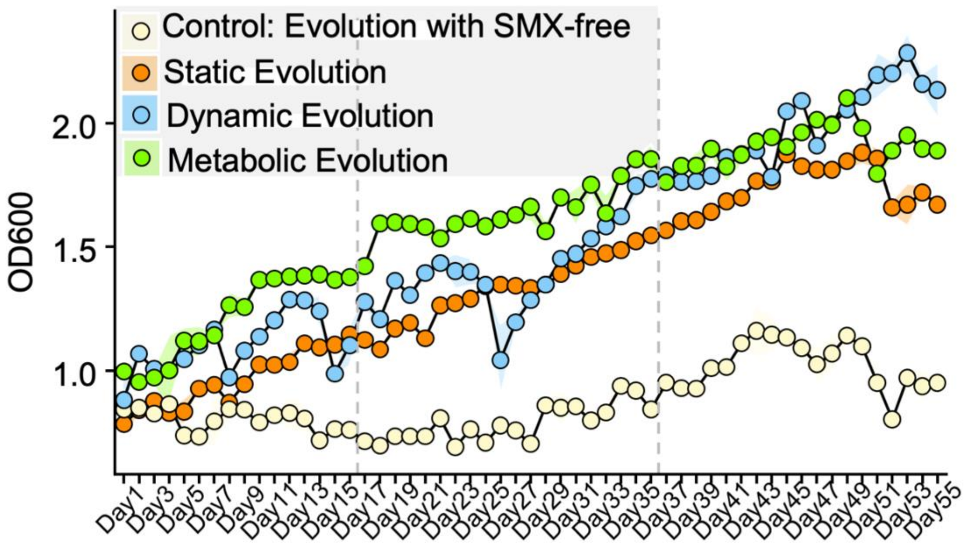

Appendix Figure S3. Growth patterns (OD_{600}) were observed over 550 generations of *C. testosteroni* evolution under static, dynamic, and metabolic evolution protocols. Each evolutionary scheme comprised six biological replicates.

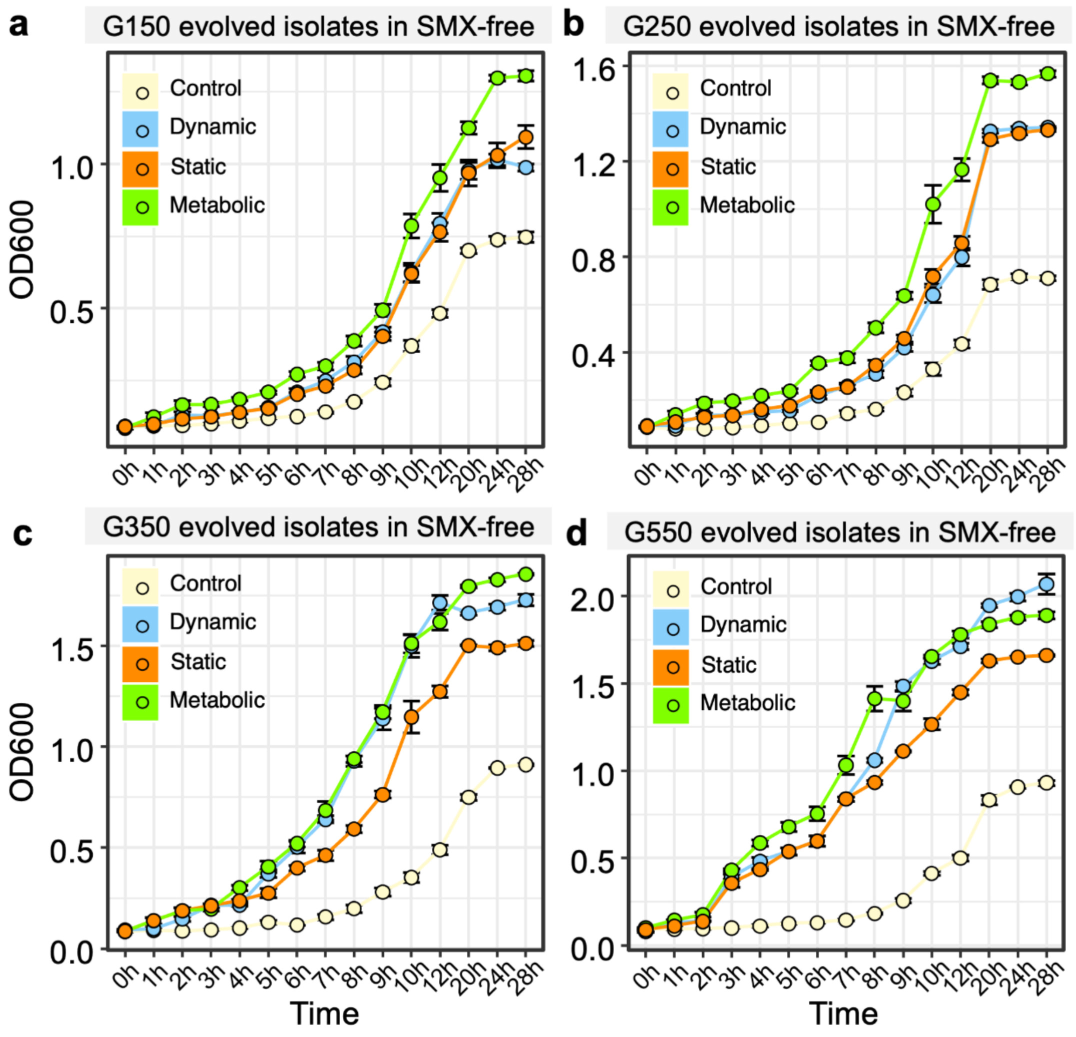

Appendix Figure S4. Growth curve was measured to determine the per capita growth rate of (**a**) G150, (**b**) G250, (**c**) G350 and (**d**) G550 evolved isolates in sulfamethoxazole (SMX)-free conditions. Time series for the optical density (OD_{600}) of each condition is shown, comprising six biological replicates.

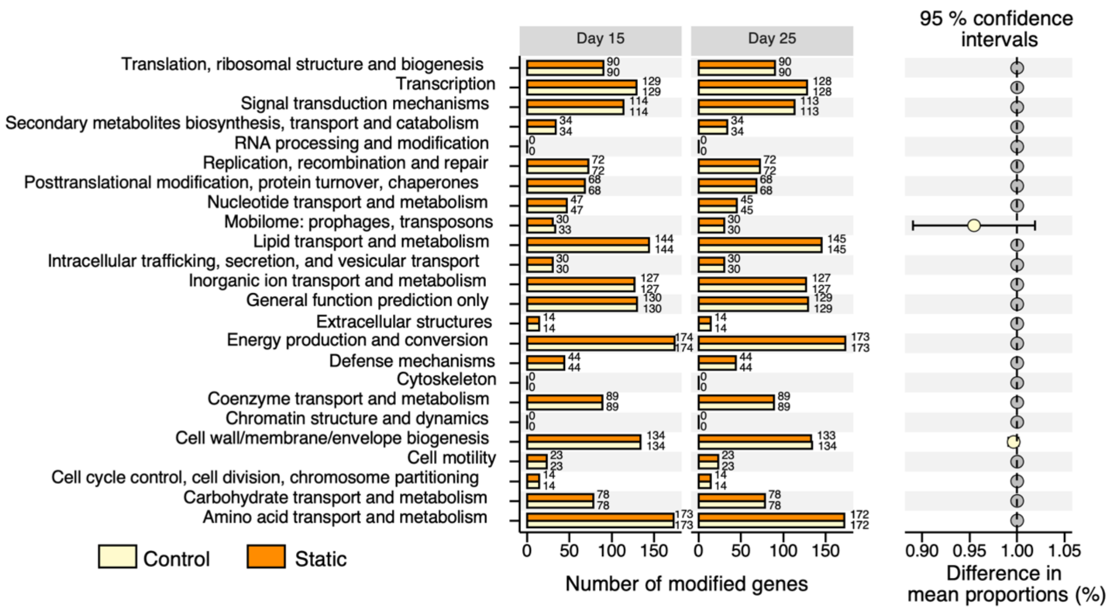

Appendix Figure S5. COG pathway enrichment analysis of bacterial methylation genes modified under static evolution. Numbers indicate quantity of methylated genes within the pathway.

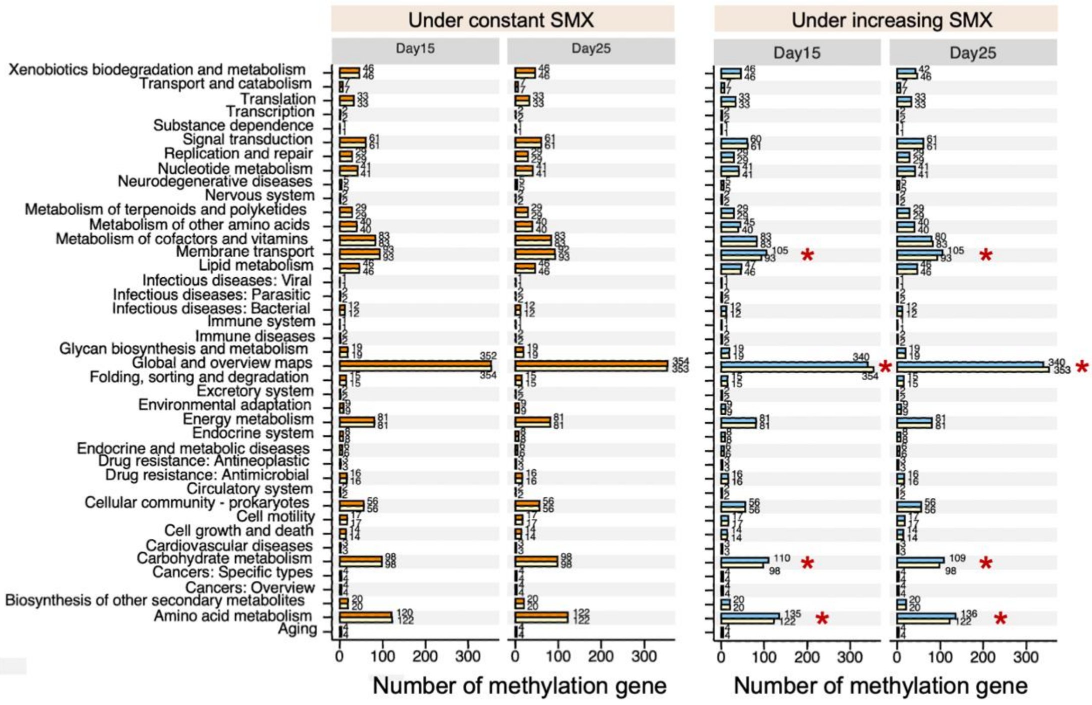

Appendix Figure S6. KEGG pathway enrichment analysis of bacterial methylation genes modified under static and dynamic evolution. Numbers indicate quantity of methylated genes within the pathway. Significance levels (*$p < 0.05$) were determined using the Wilcoxon test.

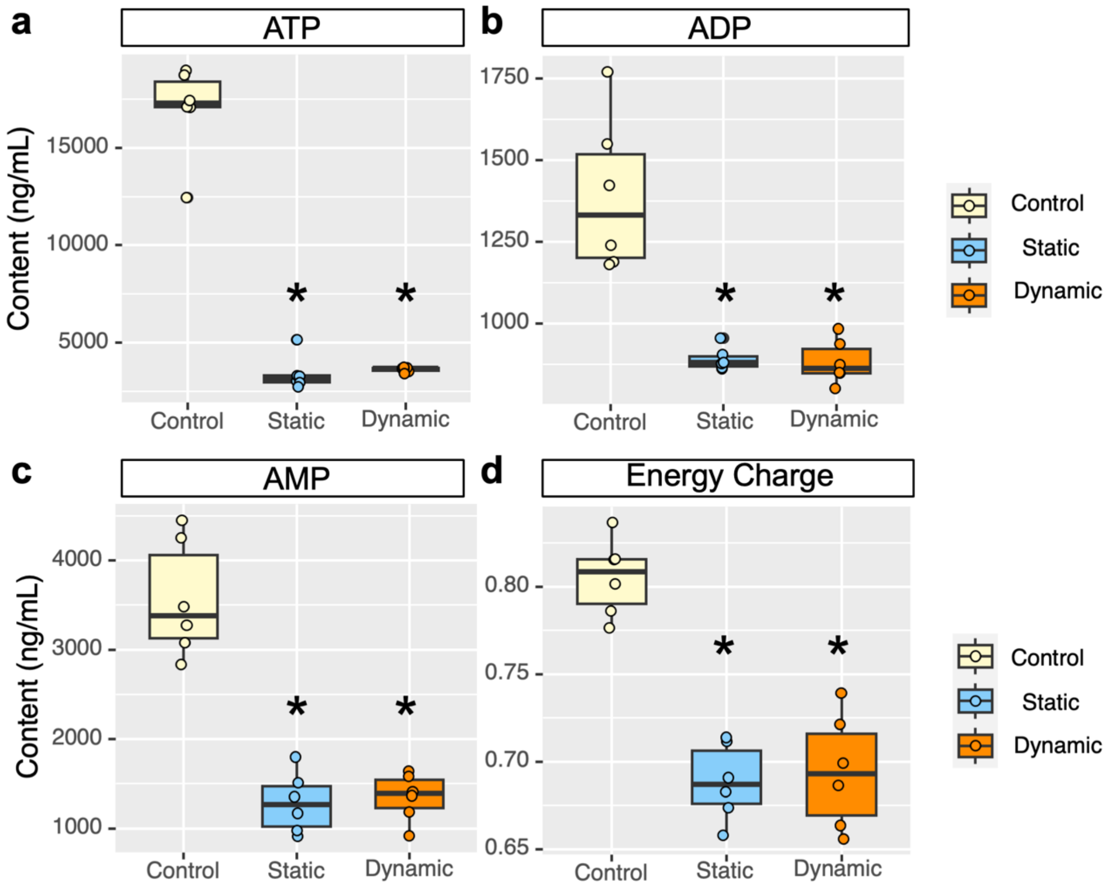

Appendix Figure S7. Analysis of evolved G550 isolates showing the content of (**a**) ATP, (**b**) ADP, and (**c**) AMP, along with (**d**) the calculated energy charge. Data are expressed as mean ± cumulative SD of six biological replicates. Significance levels (**p* < 0.05) were assessed using the Wilcoxon test.

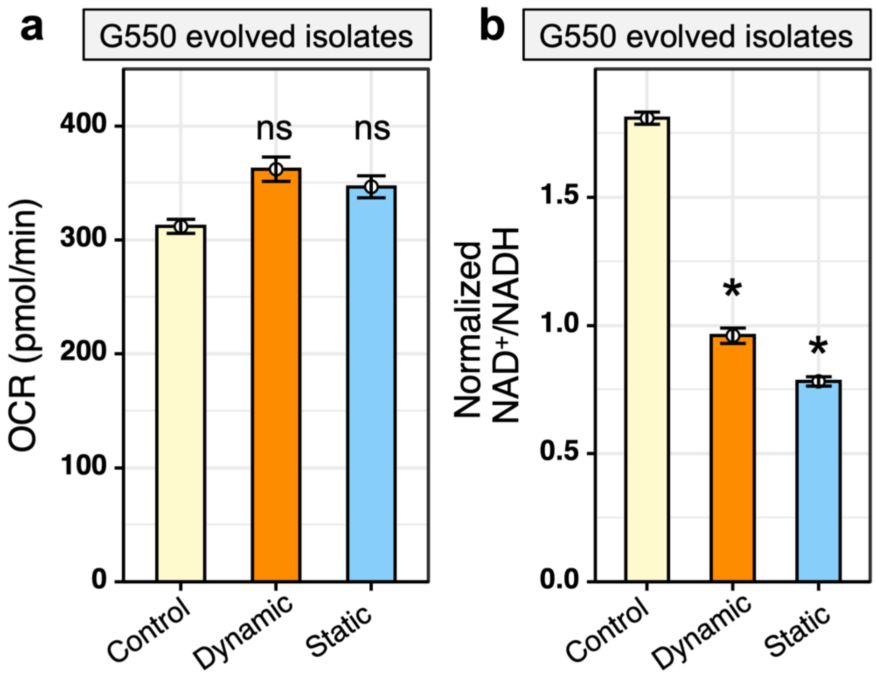

Appendix Figure S8. Physiological characteristics of G550 evolved isolates. (**a**) oxygen consumption rate (OCR) and (**b**) [NAD^+]/[NADH] redox ratio. Each measurement was biologically replicated three times.

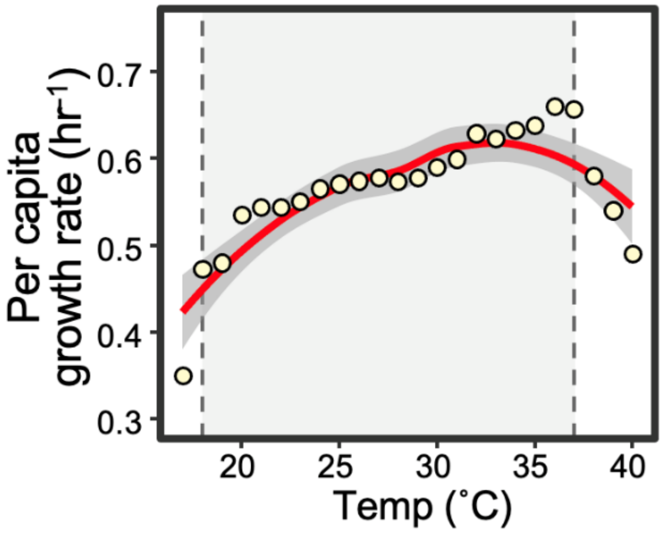

Appendix Figure S9. The growth rate response across a temperature range of 17°C to 40°C. Each data point represents the mean of six biological replicates.

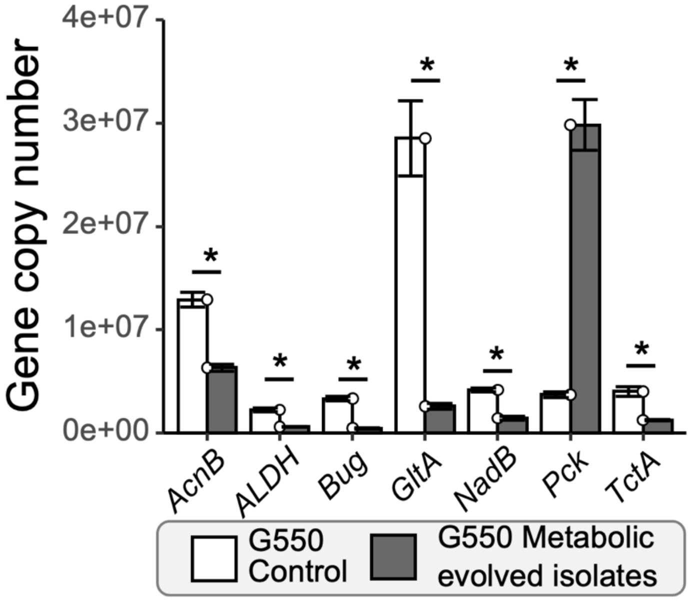

Appendix Figure S10. Assessment of metabolic gene expression in metabolically evolved resistant G550 isolates through qRT-PCR analysis. Data represents the mean ± standard deviation (SD) from three biological and three technical replicates.

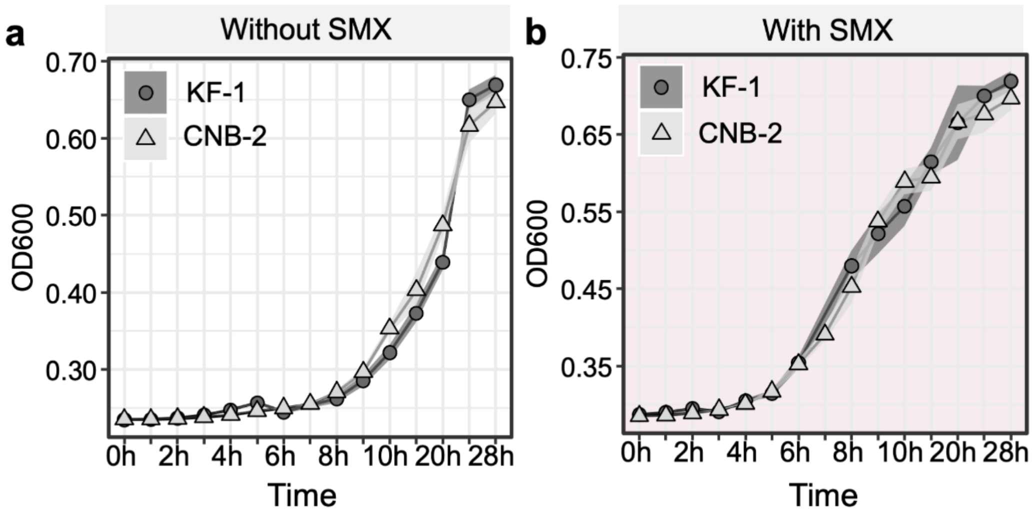

Appendix Figure S11. Growth curves for *Comamonas testosteroni* CNB-2 and KF-1 strains were measured under (**a**) SMX-free conditions and (**b**) 200 μg·L^{-1} SMX to determine their growth rates. The time series of optical density for each species in monoculture is presented with six biological replicates.

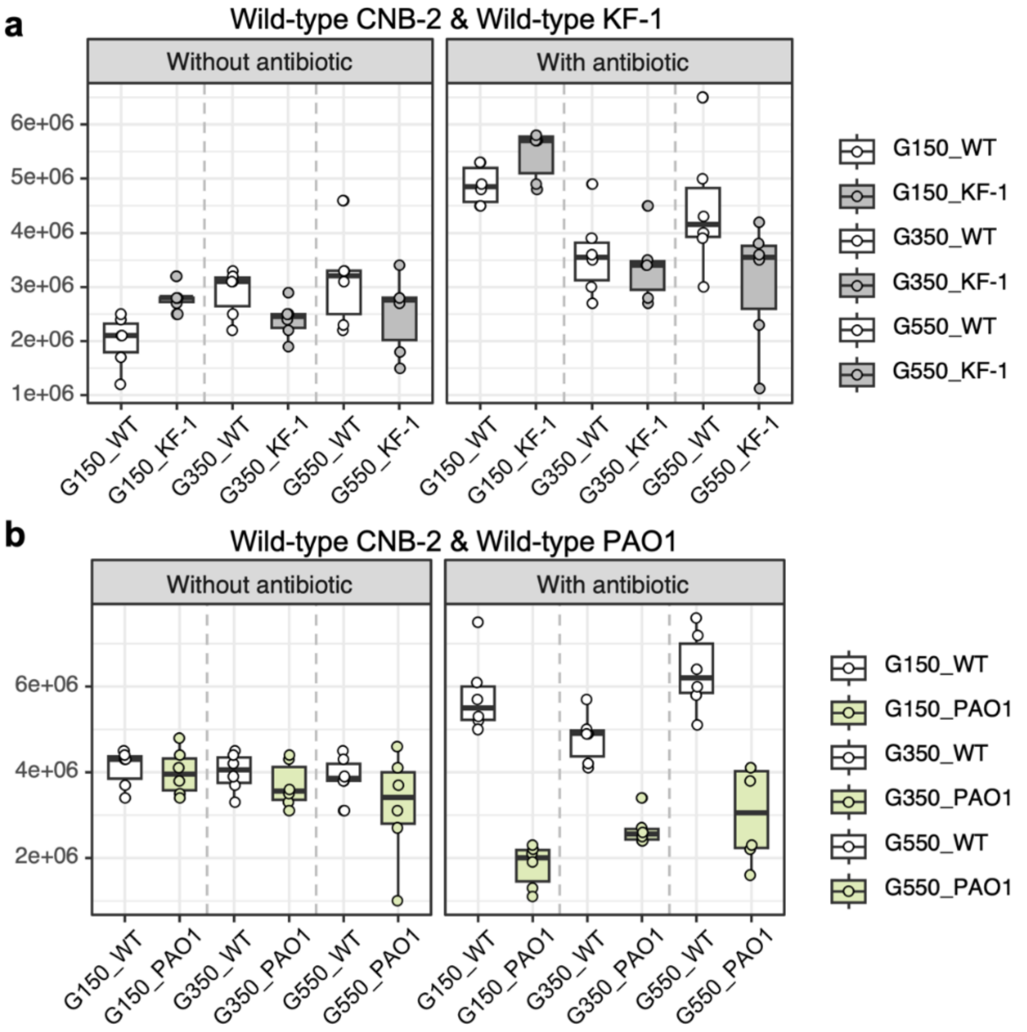

Appendix Figure S12. Competition assays between (**a**) *Comamonas testosteroni* CNB-2 wild-type and kin bacteria KF-1, or (**b**) *C. testosteroni* CNB-2 wild-type and out-group species *Pseudomonas aeruginosa* PAO1. Data represents mean ± standard deviation (SD) from six biological replicates.

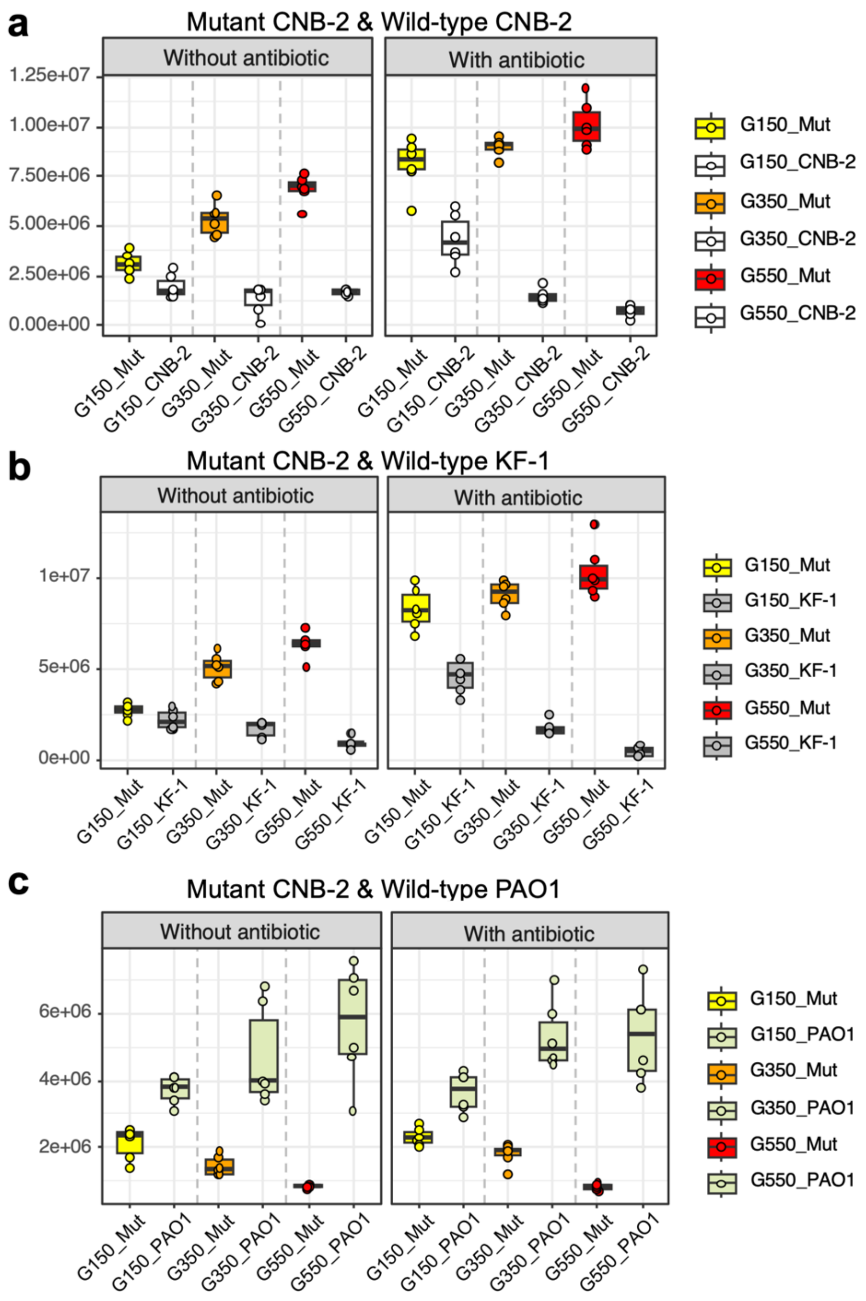

Appendix Figure S13. Competition assays between (**a**) *C. testosteroni* CNB-2 mutants and wild-type *C. testosteroni* CNB-2, (**b**) *C. testosteroni* CNB-2 mutants and kin bacteria KF-1, or (c) *C. testosteroni* CNB-2 mutants and out-group species *P. aeruginosa* PAO1. CNB-2 mutant strain displayed high intraspecific fitness but was outcompeted by PAO1 due to low interspecific fitness. Data represents mean ± standard deviation (SD) from six biological replicates.

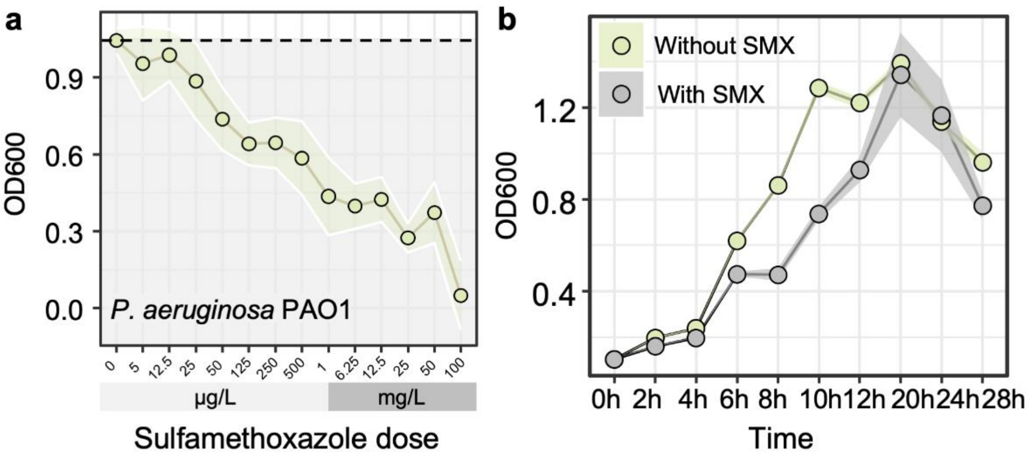

Appendix Figure S14. Antibiotic susceptibility and growth dynamics of *Pseudomonas aeruginosa* PAO1. (**a**) Antibiotic susceptibility to sulfamethoxazole (SMX) expressed as optical density at 600 nm (OD_{600}), measured after 24 hours of growth in 96-well microtiter plates. Data represents mean ± standard deviation (SD) from six biological replicates, with the horizontal dashed line indicating the average cell density observed in the absence of SMX. (**b**) Growth curve showing the per capita growth rate of *P. aeruginosa* PAO1 at 200 $\mu g \cdot L^{-1}$ SMX, used in interspecific fitness test.

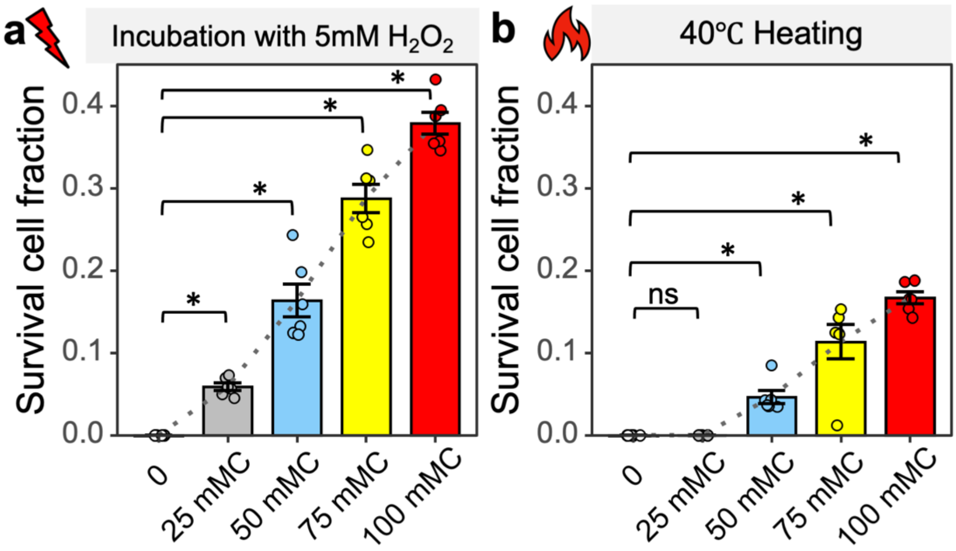

Appendix Figure S15. Survival of evolved resistant cells under (**a**) oxidative stress conditions (induced by H_2O_2) and (**b**) heat shock in NB medium supplemented with varying concentrations of succinate. Data represents mean ± standard deviation (SD) from six biological replicates.

Appendix Table S1. Per capita growth rate of *Comamonas testosteroni* cells to varying concentrations of sulfamethoxazole (SMX) during evolution on days 15 (G150), 25 (G250), 35 (G350), and 55 (G550). Red represents the ZEP range under the evolutionary scenario.

SMX concentration (mg·L^{-1})		0	0.005	0.0125	0.025	0.05	0.125	**0.25**	0.5	1	6.25	12.5	25	50	100
**Ancestral strain**		0.51	0.53	0.55	0.58	0.61	0.57	0.55	0.48	0.43	0.35	0.18	0.12	0	0
**Control: Evolution with SMX-free**	**G150**	0.55	0.58	0.60	0.61	0.64	0.65	0.6	0.51	0.45	0.43	0.21	0.15	0.05	0.04
	**G250**	0.51	0.53	0.55	0.57	0.63	0.53	0.52	0.51	0.41	0.4	0.31	0.18	0.09	0.01
	**G350**	0.53	0.55	0.58	0.59	0.64	0.60	0.53	0.49	0.42	0.35	0.20	0.11	0	0
	**G550**	0.56	0.59	0.61	0.62	0.68	0.64	0.63	0.54	0.49	0.38	0.19	0.14	0.08	0
**SMX concentration (mg·L^{-1})**		0	0.005	0.0125	0.025	0.05	0.125	0.25	0.5	1	**6.25**	**12.5**	**25**	50	100
**Static Evolution**	**G150**	0.64	0.65	0.66	0.7	0.72	0.74	0.72	0.72	0.68	0.64	0.56	0.30	0.21	0.10
	**G250**	0.64	0.66	0.66	0.71	0.73	0.75	0.70	0.69	0.67	0.65	0.55	0.34	0.25	0.14
	**G350**	0.70	0.72	0.73	0.75	0.75	0.76	0.75	0.75	0.77	0.75	0.71	0.32	0.32	0.16
	**G550**	0.80	0.80	0.81	0.83	0.85	0.85	0.87	0.85	0.83	0.82	0.81	0.84	0.49	0.29
**SMX concentration (mg·L^{-1})**		0	0.005	0.0125	0.025	0.05	0.125	0.25	0.5	1	6.25	12.5	**25**	**50**	100
**Dynamic Evolution**	**G150**	0.65	0.66	0.67	0.66	0.68	0.73	0.75	0.77	0.75	0.71	0.69	0.65	0.28	0.17
	**G250**	0.63	0.64	0.66	0.674	0.67	0.74	0.75	0.78	0.76	0.73	0.70	0.68	0.36	0.25
	**G350**	0.76	0.78	0.79	0.80	0.81	0.80	0.82	0.80	0.79	0.78	0.77	0.76	0.46	0.29
	**G550**	0.81	0.82	0.83	0.84	0.85	0.86	0.87	0.88	0.86	0.85	0.85	0.82	0.81	0.57
**SMX concentration (mg·L^{-1})**		0	0.005	0.0125	0.025	0.05	0.125	0.25	0.5	1	6.25	12.5	**25**	**50**	100
**Metabolic Evolution**	**G150**	0.67	0.72	0.74	0.73	0.75	0.81	0.83	0.85	0.82	0.78	0.76	0.72	0.31	0.19
	**G250**	0.69	0.70	0.73	0.74	0.74	0.81	0.82	0.86	0.85	0.80	0.77	0.75	0.60	0.28
	**G350**	0.74	0.86	0.87	0.88	0.89	0.88	0.80	0.88	0.87	0.76	0.85	0.84	0.71	0.32
	**G550**	0.89	0.90	0.91	0.92	0.94	0.95	0.96	0.97	0.95	0.94	0.96	0.90	0.89	0.63

Appendix Table S2 Increased methylated genes in carbohydrate transport and metabolism pathways.

Modified type	Site	Protein ID	Description	E-value
m4C	3566156	WP.012839073.1	Acetaldehyde dehydrogenase (acetylating)	5.10E-112
m4C	1171548	WP.003064341.1	Acyl-CoA reductase or other NAD-dependent aldehyde dehydrogenase	3.50E-120
m4C	257138	WP.012836707.1	2-Methylcitrate dehydratase PrpD	7.89E-215
m4C	257580	WP.012836707.1	2-Methylcitrate dehydratase PrpD	1.21E-159
m5C	5334127	WP.012840330.1	3-Phosphoglycerate kinase	2.62E-209
m4C	5334969	WP.012840330.1	3-Phosphoglycerate kinase	3.09E-286
m4C	3166475	WP.003054189.1	5-Carboxyvanillate decarboxylase LigW amidohydro domain	1.11E-36
m4C	50943	WP.012836561.1	5-Methylthioribulose/5-deoxyribulose/Fuculose 1-phosphate aldolase	1.17E-110
m4C	903185	WP.003065990.1	ABC-type glycerol-3-phosphate transport system periplasmic component	2.28E-291
m4C	899452	WP.003065985.1	ABC-type sugar transport system ATPase component MalK	1.23E-164
m5C	564398	WP.012836896.1	Beta-phosphoglucomutase HAD superfamily	1.80E-291
m4C	3876064	WP.041744268.1	Citrate lyase beta subunit	1.69E-134
m4C	3876381	WP.041744268.1	Citrate lyase beta subunit	2.91E-205
m4C	3876655	WP.041744268.1	Citrate lyase beta subunit	3.81E-185
m4C	829548	WP.012837076.1	D-hexose-6-phosphate mutarotase	1.20E-239
m4C	3920338	WP.012839282.1	Dihydroxyacid dehydratase/phosphogluconate dehydratase	4.67E-297
m4C	4435608	WP.012839673.1	Dihydroxyacid dehydratase/phosphogluconate dehydratase	2.52E-97
m4C	5029113	WP.003068405.1	DNA-binding transcriptional regulator of sugar metabolism DeoR/GlpR family	9.19E-141
m4C	3236339	WP.012838796.1	Glucose dehydrogenase PQQ-dependent	4.69E-135
m4C	3237415	WP.012838796.1	Glucose dehydrogenase PQQ-dependent	0
m4C	5032097	WP.012840095.1	Glycerol uptake facilitator or related aquaporin	2.07E-50

m4C	3264695	WP.012838812.1	H+/gluconate symporter GntT or related permease	6.16E-158
m4C	4431015	WP.012839668.1	MFS family permease includes anhydromuropeptide permease AmpG	4.08E-289
m4C	4431866	WP.012839668.1	MFS family permease includes anhydromuropeptide permease AmpG	4.21E-46
m4C	3860213	WP.012839239.1	Peptidoglycan/xylan/chitin deacetylase PgdA/NodB/CDA1 family	7.70E-179
m4C	4216630	WP.012839520.1	Peptidoglycan/xylan/chitin deacetylase PgdA/NodB/CDA1 family	6.11E-215
m4C	2663276	WP.012838302.1	Ribose 5-phosphate isomerase	7.35E-272
m4C	987316	WP.003066146.1	Sugar phosphate permease	9.46E-242
m4C	1498575	WP.039049249.1	Sugar phosphate permease	8.21E-246
m4C	2943670	WP.041744547.1	Sugar phosphate permease	2.54E-120
m4C	3167709	WP.003067553.1	Sugar phosphate permease	3.00E-273
m4C	3364493	WP.012838898.1	Sugar phosphate permease	5.50E-139
m4C	4347445	WP.021008731.1	Sugar phosphate permease	7.08E-129
m4C	5205484	WP.041744374.1	Sugar phosphate permease	7.23E-238
m4C	5205809	WP.041744374.1	Sugar phosphate permease	1.17E-128
m4C	781816	WP.003062018.1	Transaldolase/fructose-6-phosphate aldolase	4.61E-225
m4C	596445	WP.012836914.1	TRAP-type C4-dicarboxylate transport system	2.80E-220
m4C	646794	WP.012836943.1	TRAP-type C4-dicarboxylate transport system	7.08E-120
m5C	3918674	WP.003065736.1	TRAP-type C4-dicarboxylate transport system	4.79E-296
m4C	5200552	WP.012840222.1	TRAP-type C4-dicarboxylate transport system	0
m4C	5200708	WP.012840222.1	TRAP-type C4-dicarboxylate transport system	1.01E-228

Appendix Table S3 Reduced methylated genes in cell wall/membrane/envelope biogenesis pathways.

Modified type	Protein_ID	Category	Description	E_value
m4C	WP.123974905.1	Antibiotic generic targets	Membrane carboxypeptidase/penicillin-binding protein PbpC	0
m4C	WP.012838521.1		Multicopper oxidase (includes cell division protein FtsP)	8.40E-260
m4C	WP.012838521.1		Multicopper oxidase (includes cell division protein FtsP)	0
m4C	WP.012839123.1		UDP-3-O-[3-hydroxymyristoyl] glucosamine N-acyltransferase	1.17E-110
m4C	WP.003068962.1		UDP-3-O-acyl-N-acetylglucosamine deacetylase	7.89E-215
m4C	WP.003068962.1		UDP-3-O-acyl-N-acetylglucosamine deacetylase	1.21E-159
m4C	WP.003068975.1		UDP-N-acetylmuramate-alanine ligase MurC and related ligases	2.85E-205
m4C	WP.003068975.1		UDP-N-acetylmuramate-alanine ligase MurC and related ligases	8.86E-307
m4C	WP.003068975.1		UDP-N-acetylmuramate-alanine ligase MurC and related ligases	1.80E-291
m4C	WP.012839830.1		UDP-N-acetylmuramoylalanine-D-glutamate ligase	2.80E-220
m4C	WP.012839832.1		UDP-N-acetylmuramyl pentapeptide synthase	0
m4C	WP.012839832.1		UDP-N-acetylmuramyl pentapeptide synthase	7.08E-120
m4C	WP.012839832.1		UDP-N-acetylmuramyl pentapeptide synthase	0
m4C	WP.012839833.1		UDP-N-acetylmuramyl tripeptide synthase	4.61E-225
m4C	WP.012839833.1		UDP-N-acetylmuramyl tripeptide synthase	1.20E-239
m4C	WP.012839901.1		UDP-N-acetylmuramyl pentapeptide phosphotransferase	0
m4C	WP.012202833.1		Peptidoglycan/LPS O-acetylase OafA/YrhL	0
m5C	WP.012202833.1		Peptidoglycan/LPS O-acetylase OafA/YrhL	4.90E-143
m4C	WP.012202833.1		Peptidoglycan/LPS O-acetylase OafA/YrhL	9.56E-100
m4C	WP.051517707.1		Membrane carboxypeptidase/penicillin-binding protein	0
m4C	WP.051517707.1		Membrane carboxypeptidase/penicillin-binding protein	1.23E-164

m5C	WP.012840382.1		Membrane protein insertase Oxa1/YidC/SpoIIIJ	2.28E-291
m4C	WP.011255142.1	Cell membrane permeability associated with antibiotic uptake	Outer membrane protein OmpA	0
m4C	WP.012836911.1		Outer membrane porin OmpC/OmpF/PhoE	9.46E-242
m4C	WP.080692902.1		Outer membrane porin OmpC/OmpF/PhoE	0
m4C	WP.012837017.1		Outer membrane porin OmpC/OmpF/PhoE	8.21E-246
m4C	WP.003077020.1		Outer membrane protein OmpA	7.35E-272
m4C	WP.012837734.1		Outer membrane porin OmpC/OmpF/PhoE	2.54E-120
m4C	WP.012837754.1		Outer membrane porin OmpC/OmpF/PhoE	1.11E-36
m4C	WP.003061517.1		Outer membrane protein OmpA	3.00E-273
m4C	WP.003080665.1		Outer membrane protein OmpW	5.13E-148
m4C	WP.003080665.1		Outer membrane protein OmpW	4.69E-135
m4C	WP.012838545.1		Outer membrane porin OmpC/OmpF/PhoE	0
m4C	WP.012838545.1		Outer membrane porin OmpC/OmpF/PhoE	6.16E-158
m4C	WP.012839060.1		Outer membrane porin OmpC/OmpF/PhoE	5.50E-139
m4C	WP.039050672.1		Outer membrane porin OmpC/OmpF/PhoE	7.70E-179
m4C	WP.012839298.1		Outer membrane porin OmpC/OmpF/PhoE	1.69E-134
m5C	WP.012839442.1		Outer membrane porin OmpC/OmpF/PhoE	2.91E-205
m4C	WP.034355713.1		Outer membrane porin OmpC/OmpF/PhoE	3.81E-185
m4C	WP.012839694.1		Outer membrane protein OmpA	4.79E-296
m5C	WP.012839822.1		Periplasmic protein TonB	4.67E-297
m4C	WP.012839822.1		Periplasmic protein TonB	6.11E-215
m4C	WP.012839822.1		Periplasmic protein TonB	7.08E-129
m4C	WP.012840014.1		Outer membrane porin OmpC/OmpF/PhoE	4.08E-289
m4C	WP.004340371.1		Outer membrane protein OmpW	4.21E-46
m4C	WP.012840228.1		Outer membrane porin OmpC/OmpF/PhoE	2.52E-97

m4C	WP.012836547.1	Multi-drug resistant pumps	Multidrug efflux pump subunit AcrA	9.19E-141
m4C	WP.039049369.1		Outer membrane protein TolC	2.07E-50
m4C	WP.012836666.1		Multidrug efflux pump subunit AcrA	0
m4C	WP.041743862.1		Outer membrane protein TolC	1.01E-228
m4C	WP.041743862.1		Outer membrane protein TolC	7.23E-238
m4C	WP.012836785.1		Outer membrane protein TolC	1.17E-128
m4C	WP.041743956.1		Multidrug efflux pump subunit AcrA	2.62E-209
m4C	WP.012837585.1		Outer membrane protein TolC	3.09E-286
m4C	WP.003061135.1		Multidrug efflux pump subunit AcrA	1.22E-140
m5C	WP.012837726.1		Outer membrane protein TolC	1.34E-172
m4C	WP.012838505.1		Outer membrane protein TolC	1.04E-146
m4C	WP.034408170.1		Multidrug efflux pump subunit AcrA	0
m4C	WP.034408170.1		Multidrug efflux pump subunit AcrA	0
m4C	WP.034408170.1		Multidrug efflux pump subunit AcrA	0
m4C	WP.012838278.1		Outer membrane protein TolC	4.31E-273
m5C	WP.012838278.1		Outer membrane protein TolC	2.23E-162
m4C	WP.012838339.1		Outer membrane protein TolC	0
m4C	WP.012838339.1		Outer membrane protein TolC	6.95E-234
m5C	WP.012838339.1		Outer membrane protein TolC	3.51E-121
m5C	WP.012838339.1		Outer membrane protein TolC	6.14E-94
m5C	WP.012838339.1		Outer membrane protein TolC	1.93E-212
m4C	WP.041744180.1		Outer membrane protein TolC	0
m4C	WP.041744180.1		Outer membrane protein TolC	3.92E-108
m4C	WP.012838702.1		Multidrug efflux pump subunit AcrA	3.99E-146
m4C	WP.012839099.1		Outer membrane protein TolC	1.88E-118

m4C	WP.012839543.1		Outer membrane protein TolC	4.63E-224
m4C	WP.012839543.1		Outer membrane protein TolC	0
m4C	WP.012839543.1		Outer membrane protein TolC	3.42E-53
m5C	WP.012839543.1		Outer membrane protein TolC	4.64E-187
m4C	WP.012839858.1		Multidrug efflux pump subunit AcrA	1.26E-162
m4C	WP.012839858.1		Multidrug efflux pump subunit AcrA	1.47E-169
m4C	WP.012839858.1		Multidrug efflux pump subunit AcrA	0
m4C	WP.012839858.1		Multidrug efflux pump subunit AcrA	0
m4C	WP.012839860.1		Outer membrane protein TolC	7.51E-301
m4C	WP.012840050.1		Outer membrane protein TolC	0
m5C	WP.012840050.1		Outer membrane protein TolC	4.66E-113
m4C	WP.012840050.1		Outer membrane protein TolC	9.00E-162
m4C	WP.012840060.1		Outer membrane protein TolC	2.58E-177
m5C	WP.012840060.1		Outer membrane protein TolC	3.44E-243
m4C	WP.012840060.1		Outer membrane protein TolC	8.31E-195
m4C	WP.012840060.1		Outer membrane protein TolC	7.11E-275

Appendix Table S4 Physiological characteristics of 550 generations of *Comamoans testosteroni* evolution under static, dynamic, and metabolic evolution protocols. Data are expressed as mean ± SD of three biological replicates.

Parameter	G550 evolved isolates		
	**Control**	**Static**	**Dynamic**
Growth rate (h^{-1}): Rate of increase in biomass	0.56 ± 0.01	0.8 ± 0.02	0.81 ± 0.01
Biomass yield: (g_{CDW}/ $g_{Substrate}$)	0.16 ± 0.04	0.52 ± 0.06	0.55 ± 0.07
Metabolic secretions (mmol/g_{CDW}/h)	Product secretion rate		
α-Ketoglutarate (α-KG)	2.87 ± 0.3	1.59 ± 0.05	1.26 ± 0.12
Citrate	1.76 ± 0.04	ND	ND
Fumarate	ND	ND	ND
Malate	ND	ND	ND
Pyruvate	5.40 ± 0.2 × 10^{-3}	ND	ND
OAA	3.74± 0.1 × 10^{-2}	ND	ND
Glutamate	7.4 ± 2.3 × 10^{-3}	ND	ND
Acetate	ND	ND	ND
Ethanol	0.56 ± 0.15	ND	ND
Lactate	ND	ND	ND

Appendix Table S5 Reversed mutations (single nucleotide polymorphisms and InDels) of metabolically evolved isolates.

Gene	Type	Descriptions	Mutation position	Nucleotide change	Codon change	Amino-acid change
TctA^{M}	SNP	substrate-binding protein	3510344	A-**G**	ATG(H)-GTG(H)	
			3510349	A-**G**	CTC(E)-CTT(K)	Glu-Lys
Pck^{M}	SNP	phosphoenolpyruvate carboxykinase	79893	G-**T**	GCC(A)-TCC(S)	Cys-Ala
			80072	T-**G**	CTT(L)-CTG(L)	
NadB^{M}	SNP	L-aspartate oxidase	4125170	G-**A**	GGC(G)-GAC(D)	Gly-Asp
GltA^{M}	SNP	citrate synthase	3888319	A-**C**	TTC(F)-TGC(C)	Phe-Thr
ALDH^{M}	SNP	aldehyde dehydrogenase	1275850	A-**G**, T-**A**	AAT(I)-GAA(F)	Phe-Ile
AcnB^{M}	SNP	aconitate hydratase	3800754	T-**G**	TTC(E)-GTC(D)	Glu-Asp

Appendix Table S6 Physiological characteristics of wild-type *Comamonas testosteroni* CNB-2 grown on succinate or gluconate. Data are expressed as mean ± SD of three biological replicates.

Parameter	Description	Succinate	Gluconate
Growth rate (h^{-1})	Rate of increase in biomass	0.76 ± 0.05	0.36 ± 0.03
Biomass yield (g_{CDW}/ $g_{Substrate}$)	Mass of cells per substrate unit	0.46 ± 0.02	0.15 ± 0.01
Metabolic secretions (mmol/g_{CDW}/h)	Product secretion rate		
α-Ketoglutarate (α-KG)		1.031 ± 0.02	2.2 ± 0.3
Citrate		ND	1.2 ± 0.04
Fumarate		ND	ND
Malate		ND	ND
Pyruvate		ND	3.1 ± 0.2 × 10^{-3}
OAA		ND	2.6 ± 0.1 × 10^{-2}
Glutamate		6.31 ± 1.5 × 10^{3}	7.4 ± 2.3 × 10^{-3}
Succinate		—	0.9 ± 0.2 × 10^{-3}
3-Phosphoglycerate (3PG)		ND	0.94 ± 0.4 × 10^{-2}
6-Phosphogluconate (6PG)		ND	1.14 ± 0.3 × 10^{-3}

Notes:
ND: Not Detected (metabolite not found under tested conditions).
Dashes (—) indicate measurements that were not applicable or not performed.

Appendix Table S7 Strains and plasmids used in this study.

Strain or plasmid	Strain	Relevant characteristics
**Strain**		
Comamonas testosteroni	CNB-2	Wild-type
	CNB-2/G150Mut	Evolved strain under control, static and dynamic protocols
	CNB-2/G250Mut	Evolved strain under control, static and dynamic protocols
	CNB-2/G350Mut	Evolved strain under control, static and dynamic protocols
	CNB-2/G550Mut	Evolved strain under control, static and dynamic protocols
	Metabolic variant	Isolated mutant carried mutations in *TctA*, *Pck*, *NadB*, *GltA*, *Bug*, *Lpd*, *ALDH*, and *AcnB*
	KF-1	Wild-type
Comamonas testosteroni **reversed mutant**	CNB-2 Metabolic variant - $TctA^{M}$	CNB-2 metabolic mutants containing reversed genes $TctA^{M}$
	CNB-2 Metabolic variant - Pck^{M}	CNB-2 metabolic mutants containing reversed genes Pck^{M}
	CNB-2 Metabolic variant - $NadB^{M}$	CNB-2 metabolic mutants containing reversed genes $NadB^{M}$
	CNB-2 Metabolic variant - $GltA^{M}$	CNB-2 metabolic mutants containing reversed genes $GltA^{M}$
	CNB-2 Metabolic variant - $ALDH^{M}$	CNB-2 metabolic mutants containing reversed genes $ALDH^{M}$
	CNB-2 Metabolic variant - $AcnB^{M}$	CNB-2 metabolic mutants containing reversed genes $AcnB^{M}$
Escherichia coli	β2155	Transconjugation donor: F' s*trA hsdS Δ(lacZ)M15 ΔdapA::erm pir::RP4(::kan from SM10)*
Pseudomonas aeruginosa	PAO1	Wild-type
**Plasmid**		
pCVD442		Suicide vector, Gm^{R}
pCVD442 - $TctA^{M}$::Gm		Vector pCVD442 containing Gm gene cassette-truncated reversed mutant gene with flanking sequences for

	generating mutant CNB-2 Metabolic variant - *TctA*^{M}
pCVD442 - *Pck*^{M}::Gm	Vector pCVD442 containing Gm gene cassette-truncated reversed mutant gene with flanking sequences for generating mutant CNB-2 Metabolic variant - *Pck*^{M}
pCVD442 - *NadB*^{M}::Gm	Vector pCVD442 containing Gm gene cassette-truncated reversed mutant gene with flanking sequences for generating mutant CNB-2 Metabolic variant - *NadB*^{M}
pCVD442 - GltA^{M}::Gm	Vector pCVD442 containing Gm gene cassette-truncated reversed mutant gene with flanking sequences for generating mutant CNB-2 Metabolic variant - *GltA*^{M}
pCVD442 - ALDH^{M}::Gm	Vector pCVD442 containing Gm gene cassette-truncated reversed mutant gene with flanking sequences for generating mutant CNB-2 Metabolic variant - *ALDH*^{M}
pCVD442 - AcnB^{M}::Gm	Vector pCVD442 containing Gm gene cassette-truncated reversed mutant gene with flanking sequences for generating mutant CNB-2 Metabolic variant - *AcnB*^{M}

Appendix Table S8 Experimental design. Sulfamethoxazole: SMX.

Time	Control		Under constant SMX		Under increasing SMX		Under increasing temp	
	Temp	SMX	Temp	SMX	Temp	SMX	Temp	SMX
Day 1	30	0	30	0.1	30	0.1	18	0.1
Day 2	30	0	30	0.1	30	0.2	18	0.1
Day 3	30	0	30	0.1	30	0.3	18	0.1
Day 4	30	0	30	0.1	30	0.4	19	0.1
Day 5	30	0	30	0.1	30	0.5	19	0.1
Day 6	30	0	30	0.1	30	0.6	19	0.1
Day 7	30	0	30	0.1	30	0.7	20	0.1
Day 8	30	0	30	0.1	30	0.8	20	0.1
Day 9	30	0	30	0.1	30	0.9	20	0.1
Day 10	30	0	30	0.1	30	1	21	0.1
Day 11	30	0	30	0.1	30	1.1	21	0.1
Day 12	30	0	30	0.1	30	1.2	21	0.1
Day 13	30	0	30	0.1	30	1.3	22	0.1
Day 14	30	0	30	0.1	30	1.4	22	0.1
Day 15	30	0	30	0.1	30	1.5	22	0.1
Day 16	30	0	30	0.1	30	1.6	23	0.1
Day 17	30	0	30	0.1	30	1.7	23	0.1
Day 18	30	0	30	0.1	30	1.8	23	0.1
Day 19	30	0	30	0.1	30	1.9	24	0.1
Day 20	30	0	30	0.1	30	2	24	0.1
Day 21	30	0	30	0.1	30	2.1	24	0.1
Day 22	30	0	30	0.1	30	2.2	25	0.1
Day 23	30	0	30	0.1	30	2.3	25	0.1
Day 24	30	0	30	0.1	30	2.4	25	0.1
Day 25	30	0	30	0.1	30	2.5	26	0.1
Day 26	30	0	30	0.1	30	2.6	26	0.1
Day 27	30	0	30	0.1	30	2.7	26	0.1
Day 28	30	0	30	0.1	30	2.8	27	0.1
Day 29	30	0	30	0.1	30	2.9	27	0.1
Day 30	30	0	30	0.1	30	3	27	0.1
Day 31	30	0	30	0.1	30	3.1	28	0.1
Day 32	30	0	30	0.1	30	3.2	28	0.1
Day 33	30	0	30	0.1	30	3.3	28	0.1
Day 34	30	0	30	0.1	30	3.4	29	0.1
Day 35	30	0	30	0.1	30	3.5	29	0.1
Day 36	30	0	30	0.1	30	3.6	29	0.1

Day 37	30	0	30	0.1	30	3.7	30	0.1
Day 38	30	0	30	0.1	30	3.8	30	0.1
Day 39	30	0	30	0.1	30	3.9	30	0.1
Day 40	30	0	30	0.1	30	4	31	0.1
Day 41	30	0	30	0.1	30	4.1	31	0.1
Day 42	30	0	30	0.1	30	4.2	31	0.1
Day 43	30	0	30	0.1	30	4.3	32	0.1
Day 44	30	0	30	0.1	30	4.4	32	0.1
Day 45	30	0	30	0.1	30	4.5	32	0.1
Day 46	30	0	30	0.1	30	4.6	33	0.1
Day 47	30	0	30	0.1	30	4.7	33	0.1
Day 48	30	0	30	0.1	30	4.8	33	0.1
Day 49	30	0	30	0.1	30	4.9	34	0.1
Day 50	30	0	30	0.1	30	5	34	0.1
Day 51	30	0	30	0.1	30	5.1	35	0.1
Day 52	30	0	30	0.1	30	5.2	35	0.1
Day 53	30	0	30	0.1	30	5.3	36	0.1
Day 54	30	0	30	0.1	30	5.4	36	0.1
Day 55	30	0	30	0.1	30	5.5	37	0.1

Appendix Table S9 Composition of mineral salt medium (MSM) used in this study.

**Chemical**	**Concentration**
Gluconate (or Succinate)	50 mM
KNO_3	10 mM
$MgSO_4$•$7H_2O$	2 mM
$CaCl_2$•$2H_2O$	0.4 mM
KH_2PO_4	4 mM
HEPES [4-(2-hydroxyethyl)-1-piperazineethanesulfonic acid]	10 mM
Vitamin B_1	0.001 $mg·L^{-1}$
Vitamin B_{12}	0.002 $mg·L^{-1}$
Biotin	0.003 $mg·L^{-1}$

Appendix Table S10 Primers used in RT-PCR.

Primer	Description	Source
AcnB-a	GCCATGCTTCGACCTTTTCG	This paper
AcnB-b	CTGGTGCATGCGATTCCCTT	This paper
TctA-a	CCGGCAAGATCACCTCGTAT	This paper
TctA-b	CGCCTTGATGTACTGGGCTA	This paper
Bug-a	TTCCCCTGGTGATGAAGCAG	This paper
Bug-b	CATAGCTTTTGACGGGCGTG	This paper
ALDH-a	CAGACCAGTTTTCCGCCCTT	This paper
ALDH-b	CATGTCCACCGAACGCTTTG	This paper
GltA-a	GCGTTCTACGCGCTTGAAAT	This paper
GltA-b	ATTGTCCATGCGGTTGTTGC	This paper
NadB-a	AGGGCGTGGAAGTCAATGTC	This paper
NadB-b	ATGGCGGATCGATTTCAGGC	This paper
Pck-a	CAGATGCCATTGCCCTTTCC	This paper
Pck-b	CAGATGCCATTGCCCTTTCC	This paper

Appendix Table S11 Targeted metabolomics analysis for exploring cellular central carbon metabolic pathway.

Category	Chemical	Abbreviation	MS detection parameters				
			Q1	Q3	RT	DP	CE
Nucleotides	CMP	CMP	185	92.2	8.99	-40	-10
	FAD	FAD	362.2	211.1	10.74	-25	-24
	$NADP^+$	$NADP^+$	810.3	427.9	9.94	58	37
	dAMP	dAMP	522	424	15.68	-30	-23
	ATP	ATP	229	139.1	10.36	-28	-19
	Adenosine diphosphate	ADP	425.9	134.1	10.67	-88	-30
	AMP	AMP	346	79	9.84	-30	-60
	GTP	GTP	522	424	15.68	-30	-23
	GMP	GMP	362.2	211.1	10.74	-25	-24
Phosphorylated intermediates	D-Glucose 1-phosphate	G1P	259.2	96.9	10.59	-68	-24
	Phosphoenol pyruvic acid	PEP	166.8	78.9	12.03	-32	-17
	Ribose 5-phosphate	R5P	228.9	79.1	10.45	-46	-44
	D-Erythrose 4-phosphate	E4P	199	97	10.97	-30	-19
	D-Xylulose 5-phosphate	Xu5P	87	42.8	7.55	-29	-11
	Sedoheptulose 7-phosphate	S7P	339	241.2	14.05	-23	-19
	D-Fructose 1,6-bisphosphate	FBP	258.9	97	10.98	-31	-20
	Glucose 6-phosphate	G6P	173	85	10.78	-50	-17
	Thiamine diphosphate	ThDP	664.2	136.3	9.89	59	42
	Dihydroxyacetone phosphate	DHAP	328.2	134	8.7	-46	-29
	3-Phospho-D-glycerate	3PG	184.8	79.1	11.34	-26	-21
	2-Phospho-D-glycerate	2PG	136	74.2	11.42	-20	-22
	NAD^+	NAD^+	810.3	427.9	9.94	58	37

	Triphosphopyridine nucleotide	NADPH	810.3	427.9	9.94	58	37
	D-Glucose	D-Glucose	114.9	71.1	10.97	-44	-11
	Cyclic AMP	CAMP	185	92.2	8.99	-40	-10
	Phosphogluconic acid	6PG	275	96.8	11.77	-46	-20
TCA cycle-related organic acids	Isocitrate	Isocitrate	191	73.2	12.19	-49	-29
	Malate	Malate	133.1	70.8	11.44	-33	-19
	Pyruvic Acid	Pyruvate	116.9	73.1	10.19	-39	-15
	Succinic Acid	Succinate	362.2	211.1	10.74	-25	-24
	Fumaric Acid	Fumarate	192.8	112.9	10.19	-26	-15
	Acetyl-CoA	Acetyl-CoA	337.1	141	9.65	76	21
	Citric Acid	Citrate	185	92.2	8.99	-40	-10
	Ketoglutaric acid	α-KG	345	211.1	10.02	42	-14
	cis-Aconitic acid	cis-Aconitic	321.2	195.1	9.47	-33	-22
	Oxaloacetate	OAA	189	82.2	9.29	-42	-15
	Lactic acid	Lactate	89	43	7.97	-30	-20

Appendix Table S12 Reversed mutations primers used in this study.

Primer	Description
**Upstream homologous recombination arm primers (-5F, 5' Phosphorylated)**	
TctA-5F	TTCACAGGAGGTGGAGTCTATG
TctA-5R	CACTCCACGCAGATCGGC
Pck-5F	CCTTCCTGGCCTGGACCG
Pck-5R	GAAATTGGTCTTGCCGCAGGC
NadB-5F	GATTTTGCCGCTGCCCAGC
NadB-5R	CGGCAGGCACAGCAAGGC
GltA-5F	GGCGACAAGGGTGAGCTG
GltA-5R	GGCTTCGTTGGCGCCGC
ALDH-5F	GGCATGTTTCTGTTCAACGGGCTGGAGAATCTGATGGG
ALDH-5R	GGCCCGGTCAAGGGCATCGTGCG
AcnB-5F	AGAGCAAAGTCGGCAAAGAACG
AcnB-5R	CGGTGCGGAGCGCAACAA
**Downstream homologous recombination arm primers (-3R, 5' Phosphorylated)**	
ALDH-3F	GCTCTCCTCGTGGAAGATGCGCATCGCC
ALDH-3R	GGCTTCGCTGACCACGCAGATCAATGGCG
TctA-3F	AGCAATGATGGGCTTGGTCCG
TctA-3R	TGACAGTCATGTTCATGATGGTTTT
Pck-3F	ATGCTGGTGCCGCCCAAG
Pck-3R	CGTACTGGGGAGCAATGCC
NadB-5F	CTATGGGCAATTGGCGAGGT
NadB-5R	TTTTTGCTTTCCAGGGTGGCG
GltA-3F	GCGGCTTCCGTCGTGACG
GltA-3R	GGCCAGAGCGAAGATGCC
AcnB-3F	CGACAGGGGTTTGCTTCTTCG
AcnB-3R	GATGCGGTGTACTACCCCGA
**Linker Primer (Red: Bases that have been substituted)**	
TctA-linker	GTGCCGCCGATCTGCGTGGAGTG**CTT**AGCAATGATGGGCTTGGTCCGCT
ALDH-linker	CGCACGATGCCCTTGACCGGGCC**AAG**CTCTCCTCGTGGAAGATGCGCAT
Pck-linker	GGCCTGCGGCAAGACCAATTTC**TCC**ATGCTGGTGCCGCCCAAGGCCTT
NadB-linker	GGAGCCTTGCTGTGCCTGCCG**GAC**CGGCACGGCCAGCCCGGCGAGCG
GltA-linker	ACGGCGGCGCCAACGAAGCC**TCG**GCGGCTTCCGTCGTGACGCTCACCCC
AcnB-linker	CTCGGCCTTGTTGCGCTCCGCACCG**GTC**CGACAGGGGTTTGCTTCTTCG

References

Deatherage DE, Barrick JE (2014) Identification of mutations in laboratory-evolved microbes from next-generation sequencing data using breseq. *Engineering analyzing multicellular systems: methods protocols*: 165-188

Koboldt DC, Chen K, Wylie T, Larson DE, McLellan MD, Mardis ER, Weinstock GM, Wilson RK, Ding L (2009) VarScan: variant detection in massively parallel sequencing of individual and pooled samples. *Bioinformatics* 25: 2283-2285

Li H, Durbin R (2009) Fast and accurate short read alignment with Burrows–Wheeler transform. *Bioinformatics* 25: 1754-1760

Martin M (2011) Cutadapt removes adapter sequences from high-throughput sequencing reads. *EMBnet journal* 17: 10-12

Wang Z-J, Liu S-S, Qu R (2018) JSFit: a method for the fitting and prediction of J-and S-shaped concentration–response curves. *RSC advances* 8: 6572-6580
