# Supplementary figures and images for: Epigenetic modifications and metabolic gene mutations drive resistance evolution in response to stimulatory antibiotics
Source: Mol Syst Biol. 2025 Jan 16;21(3):294–314. doi: 10.1038/s44320-025-00087-4 (PMC11876630; doi:10.1038/s44320-025-00087-4)

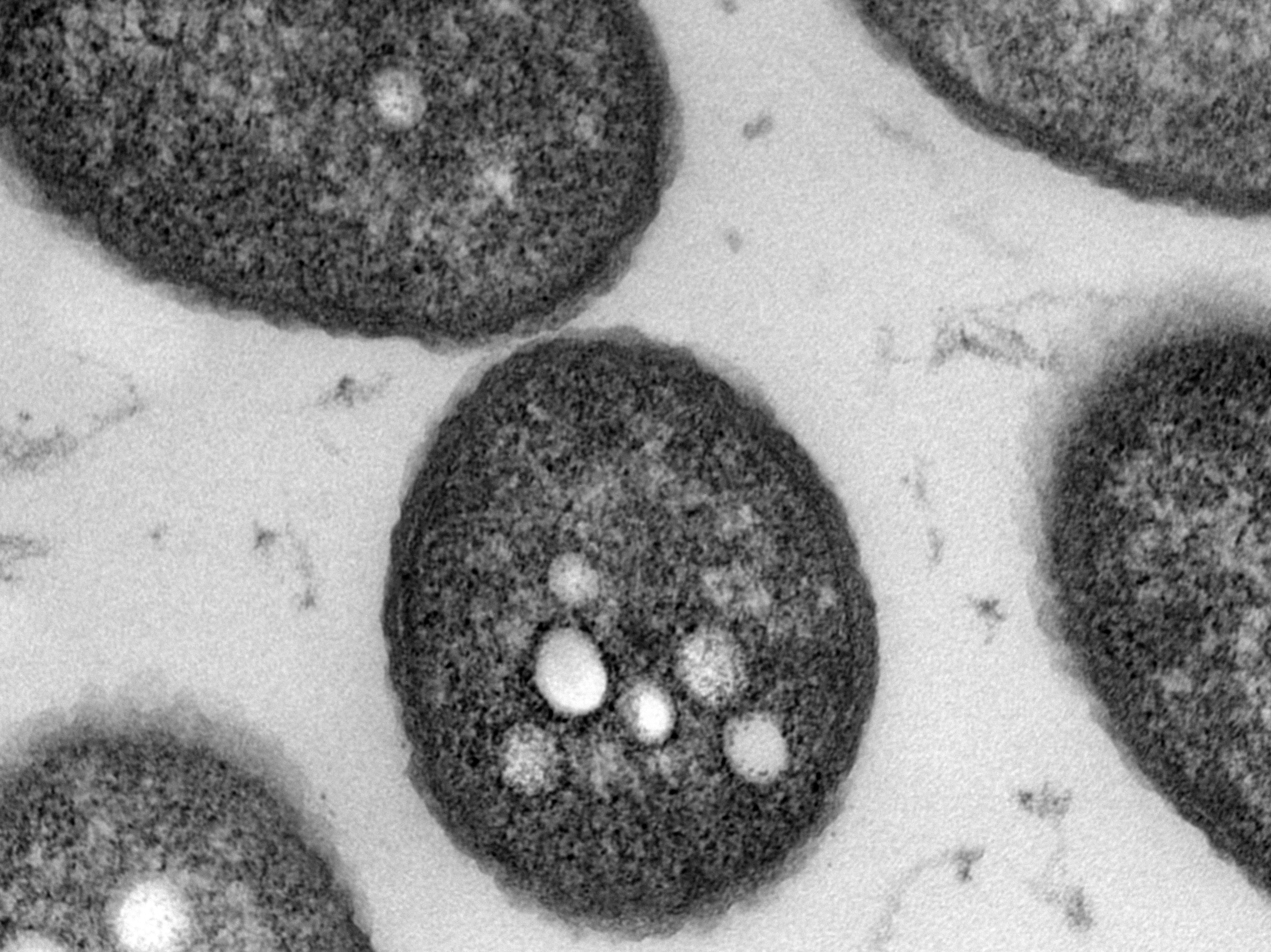

Supplement: Supplementary file 4 — Source data Fig. 2 [file 44320_2025_87_MOESM4_ESM.zip › Figure 2/2d/Dynamic-40k.tif]

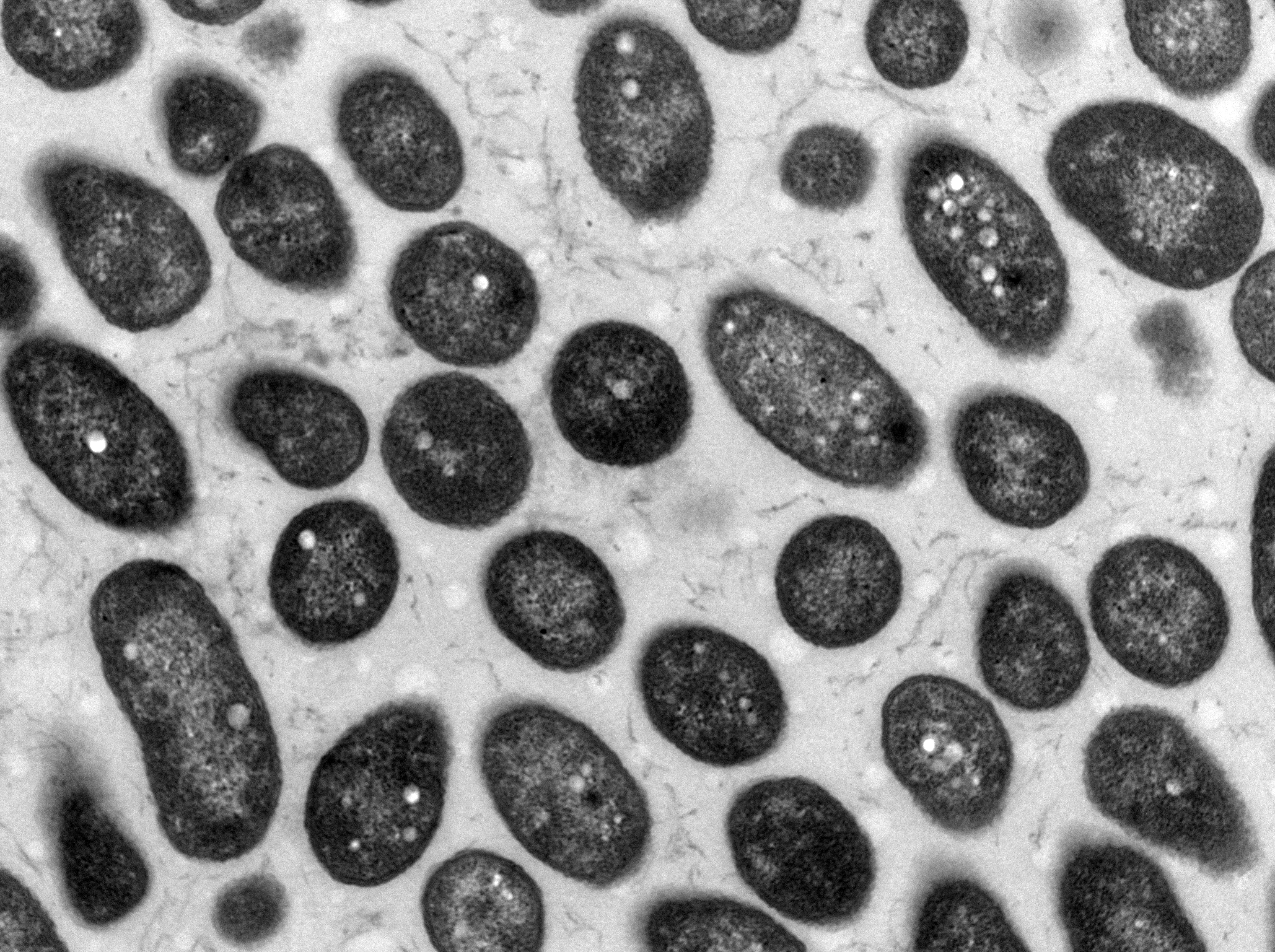

Supplement: Supplementary file 4 — Source data Fig. 2 [file 44320_2025_87_MOESM4_ESM.zip › Figure 2/2d/Dynamic-10k.tif]

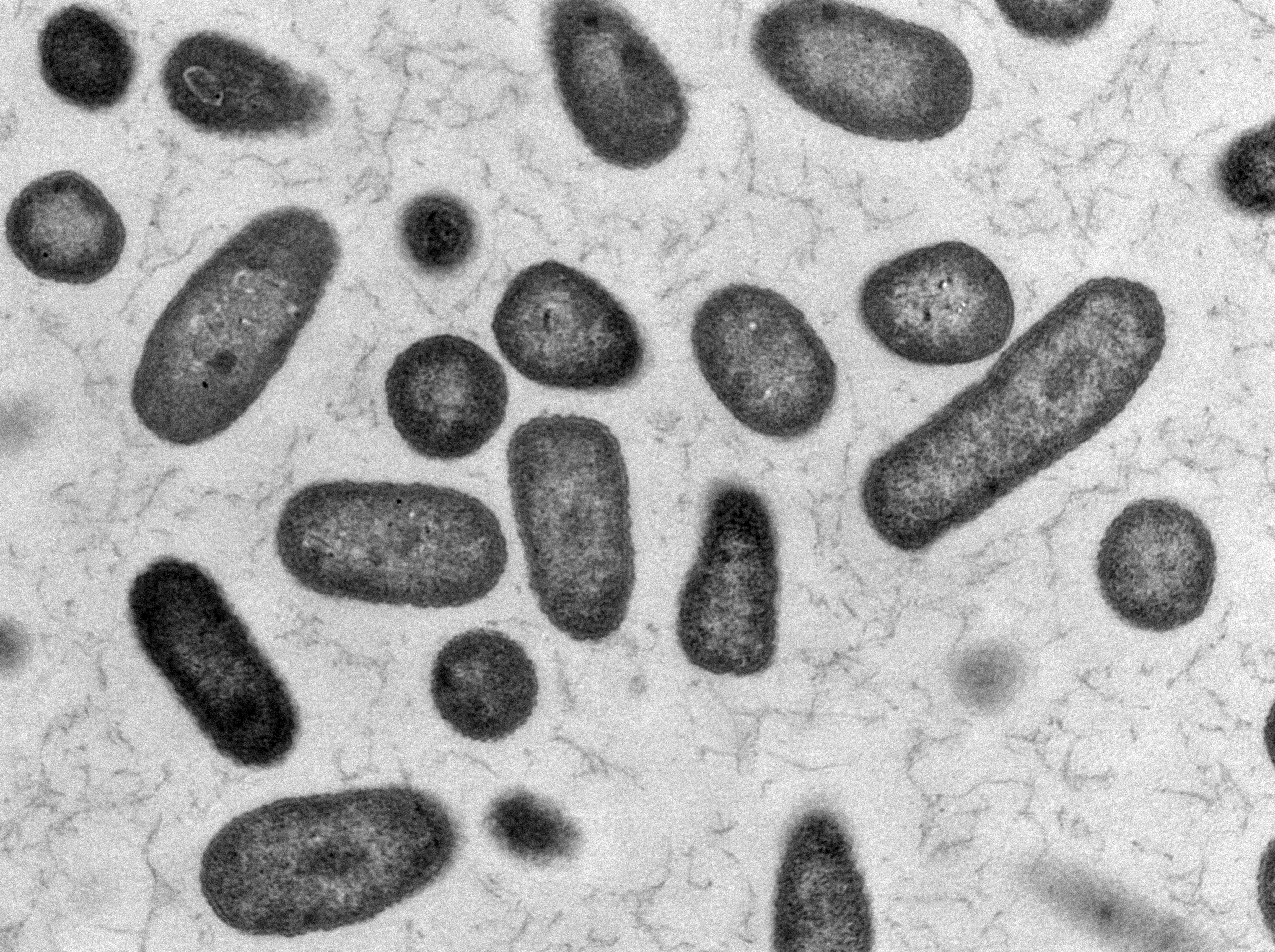

Supplement: Supplementary file 4 — Source data Fig. 2 [file 44320_2025_87_MOESM4_ESM.zip › Figure 2/2d/Control-10k.tif]

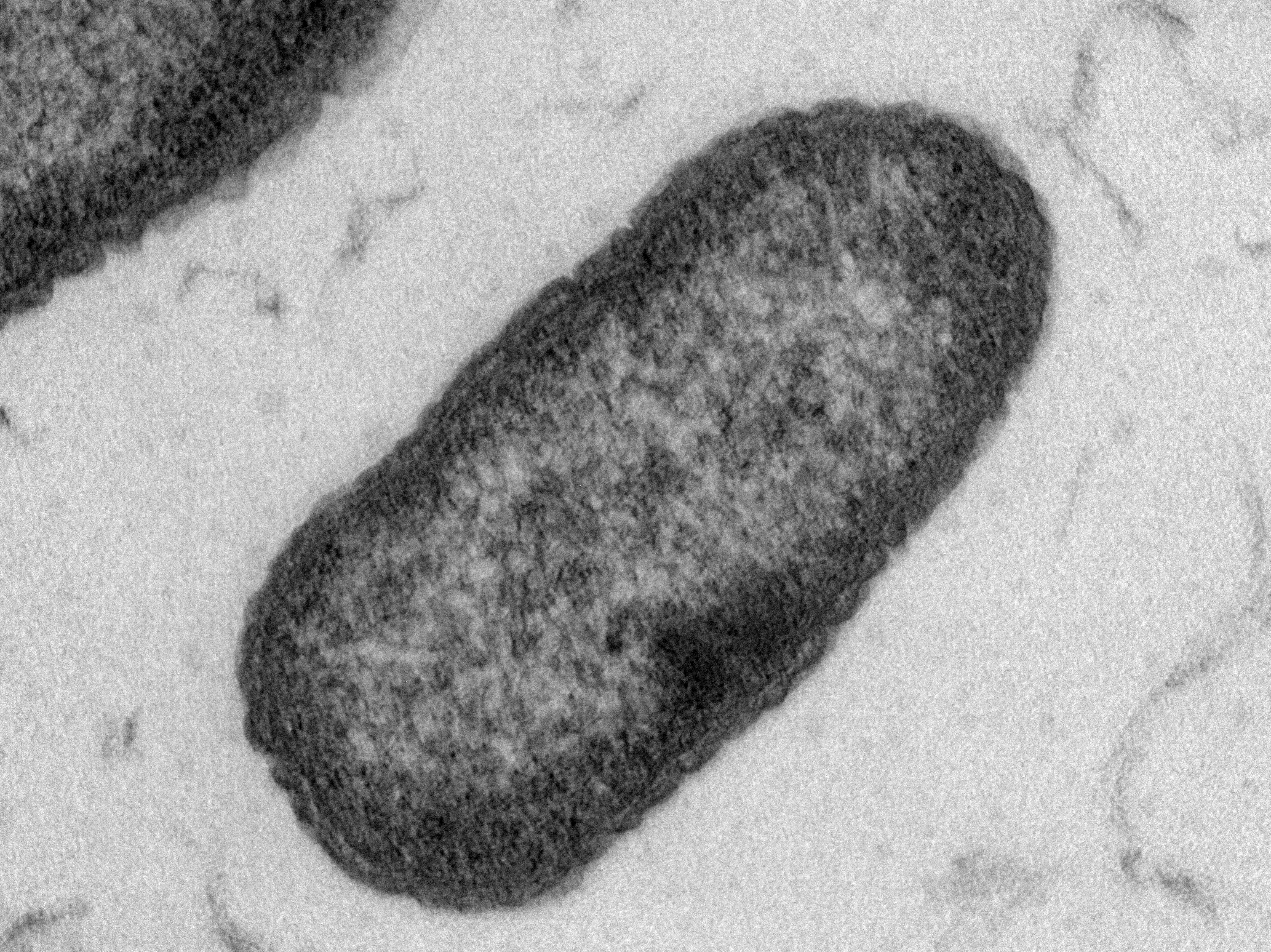

Supplement: Supplementary file 4 — Source data Fig. 2 [file 44320_2025_87_MOESM4_ESM.zip › Figure 2/2d/Static-40k.tif]

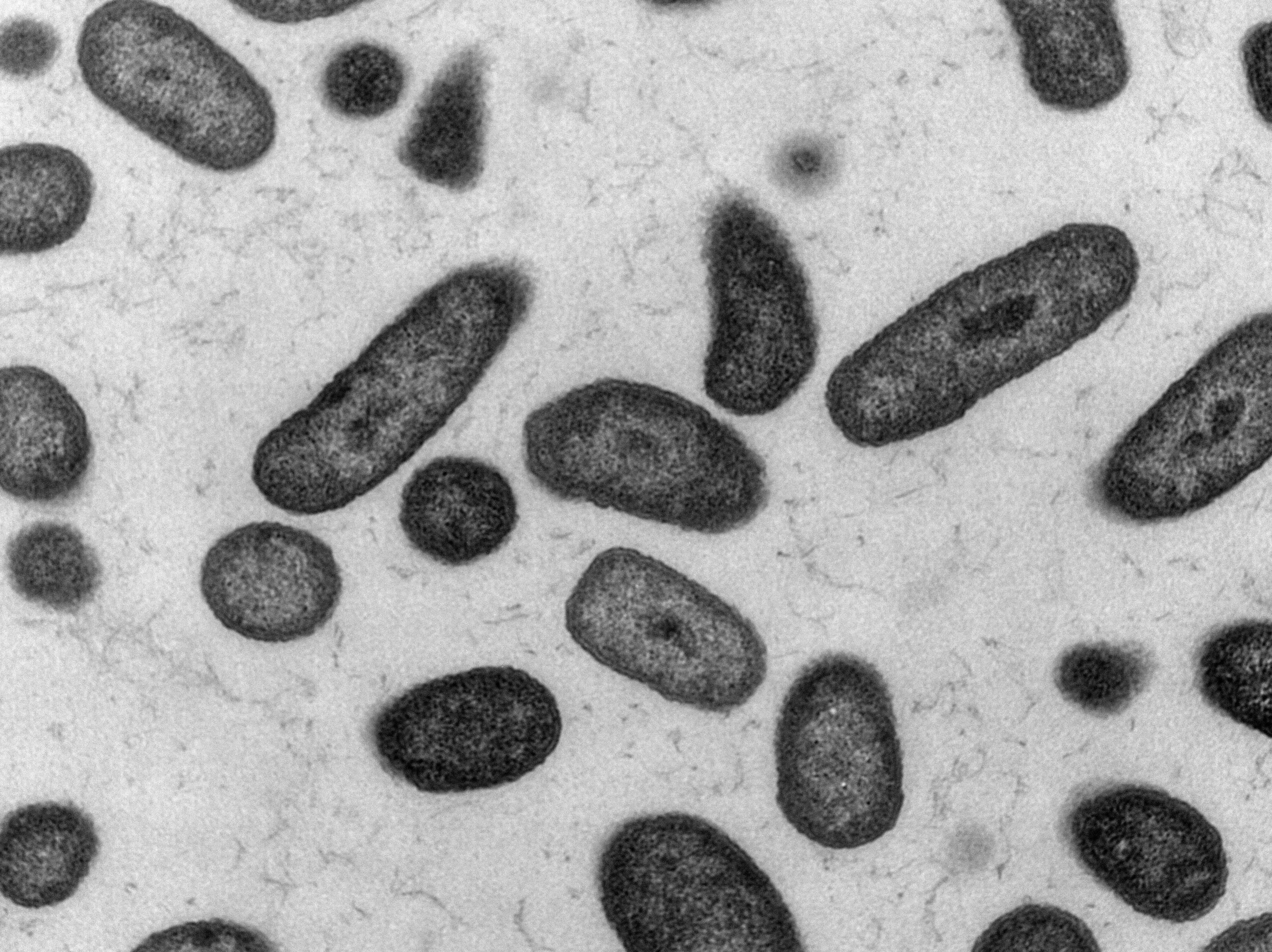

Supplement: Supplementary file 4 — Source data Fig. 2 [file 44320_2025_87_MOESM4_ESM.zip › Figure 2/2d/Static-10k.tif]

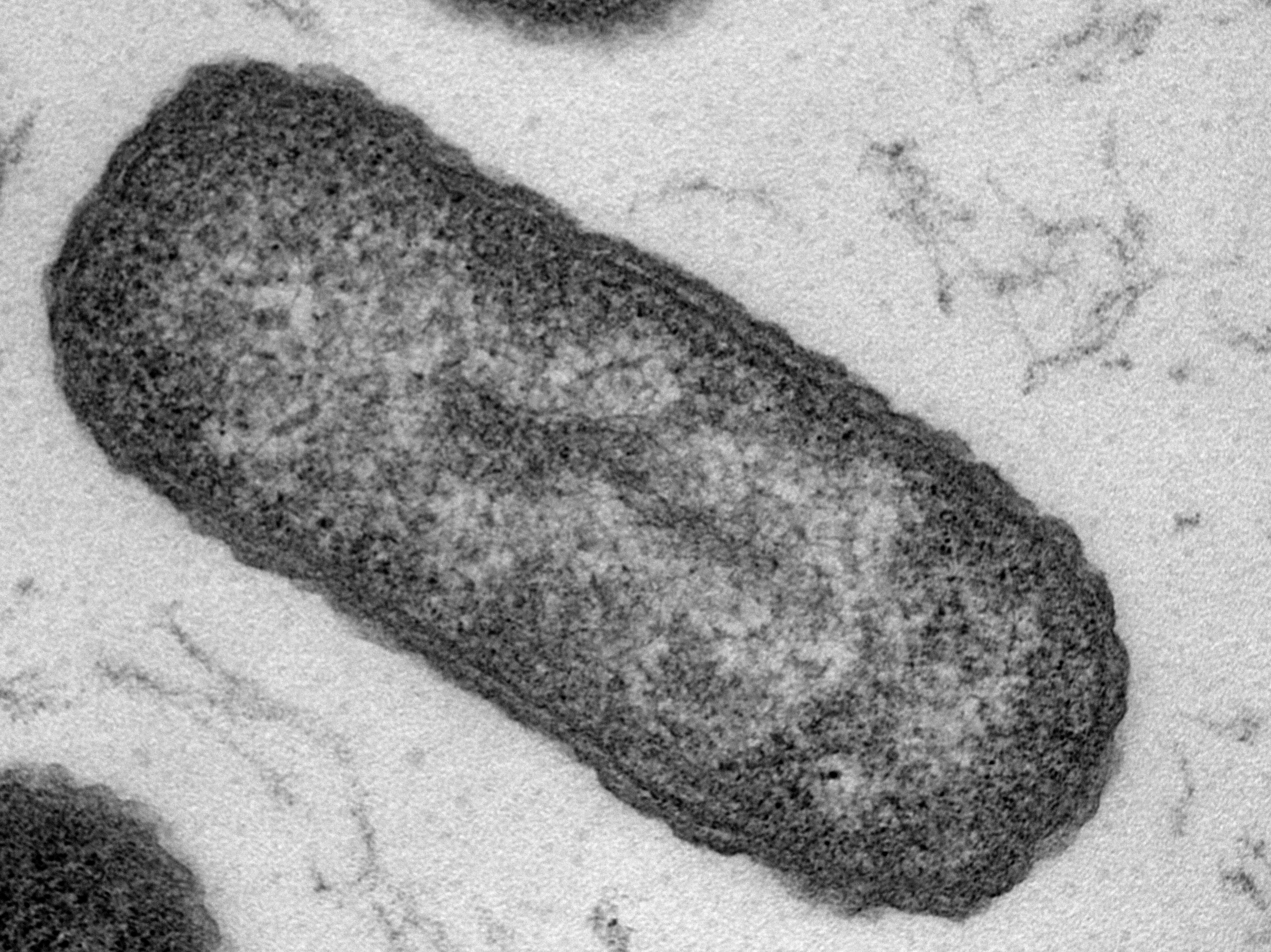

Supplement: Supplementary file 4 — Source data Fig. 2 [file 44320_2025_87_MOESM4_ESM.zip › Figure 2/2d/Control-40k.tif]

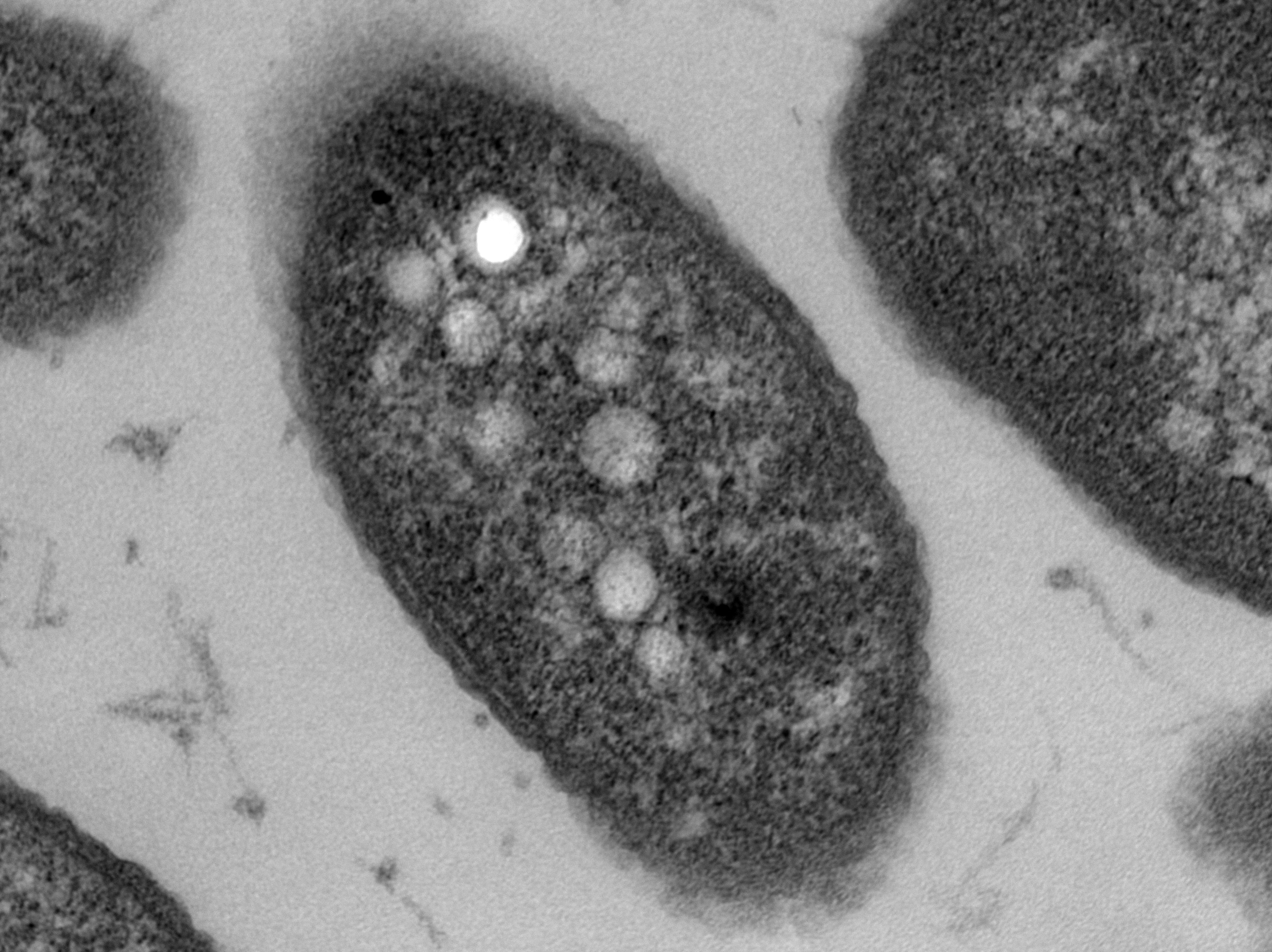

Supplement: Supplementary file 7 — Source data Fig. 5 [file 44320_2025_87_MOESM7_ESM.zip › Figure 5/5c/Suc-40k.tif]

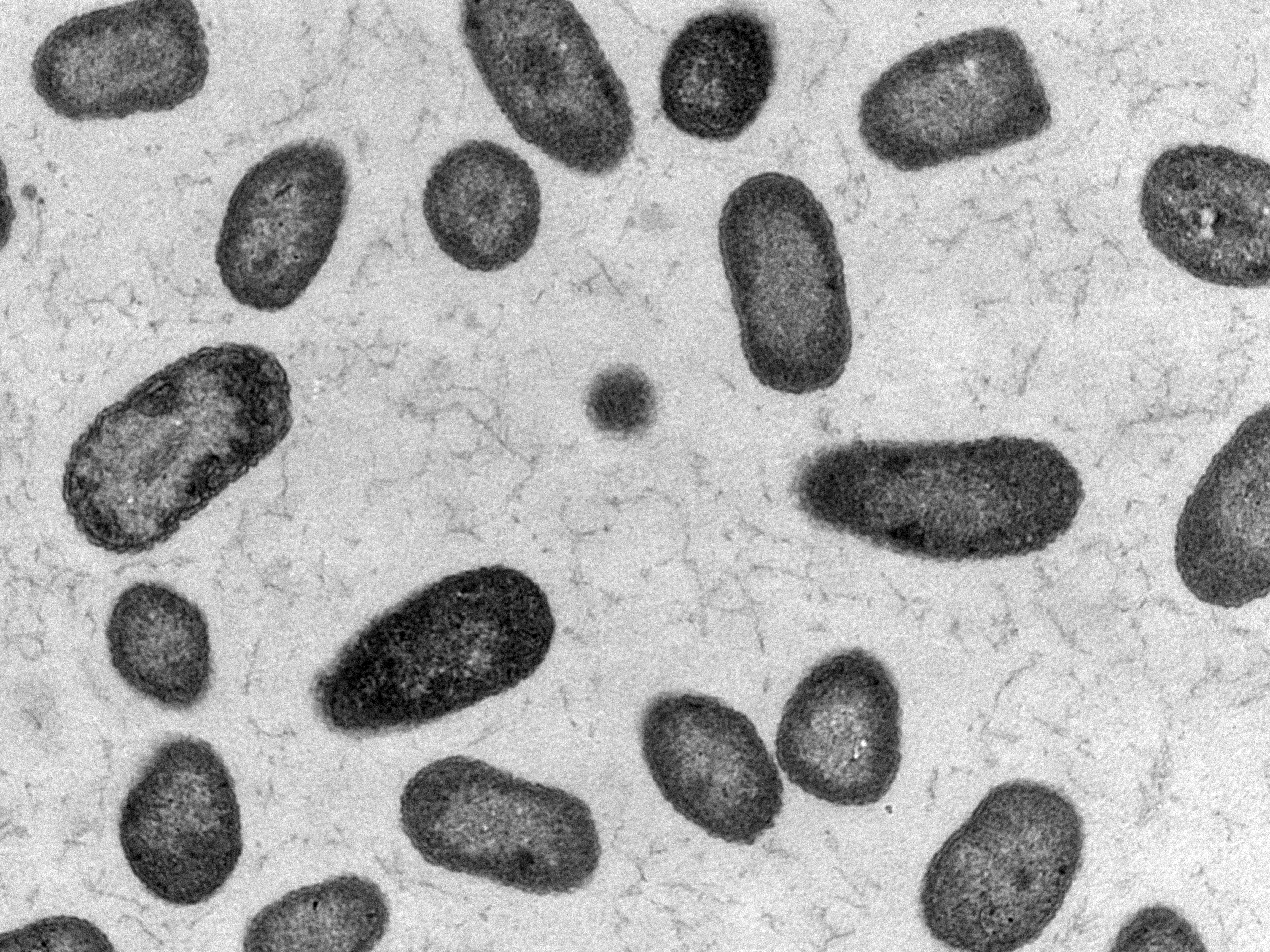

Supplement: Supplementary file 7 — Source data Fig. 5 [file 44320_2025_87_MOESM7_ESM.zip › Figure 5/5c/Glu-10k.tif]

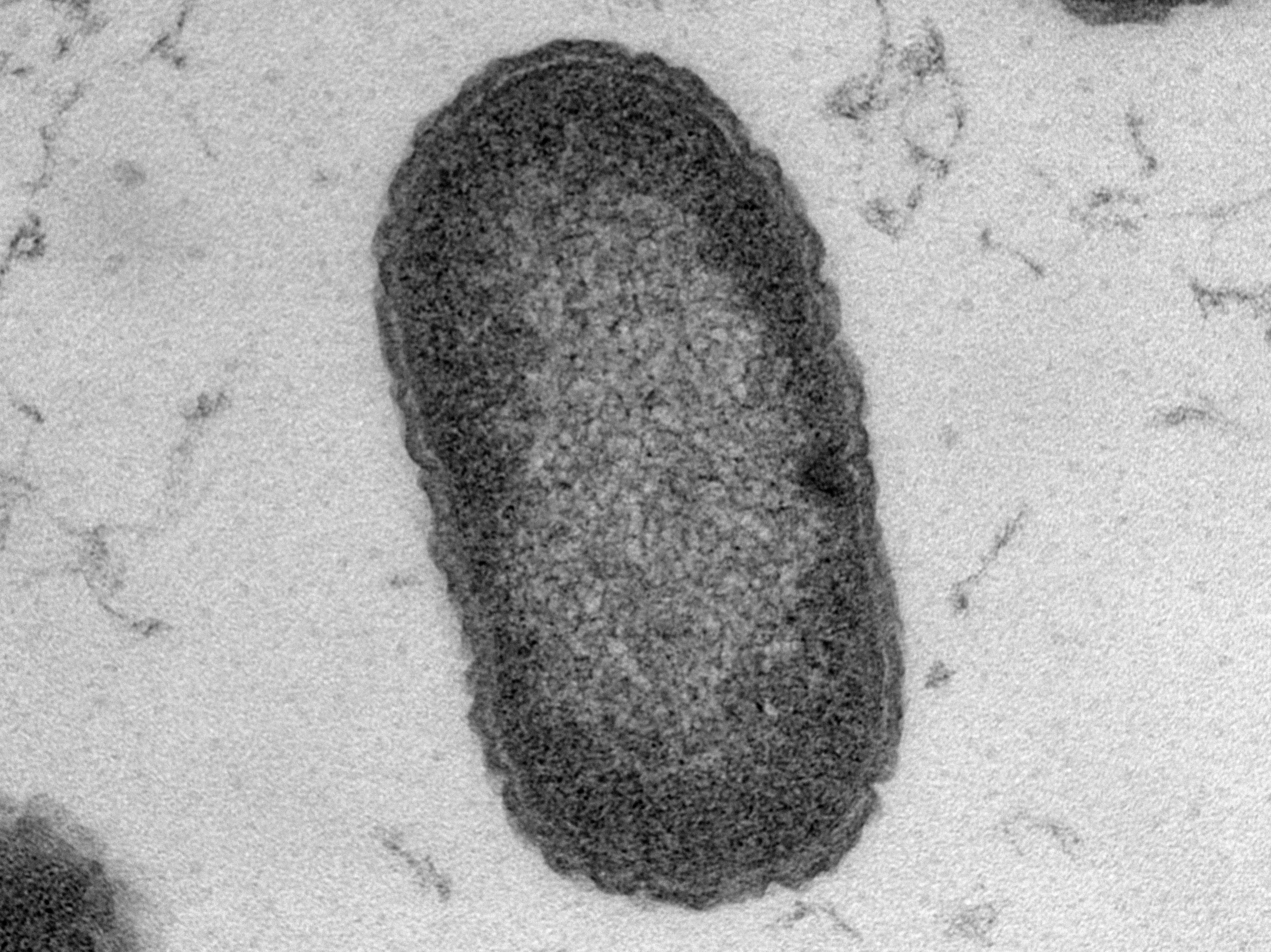

Supplement: Supplementary file 7 — Source data Fig. 5 [file 44320_2025_87_MOESM7_ESM.zip › Figure 5/5c/Glu-40k.tif]

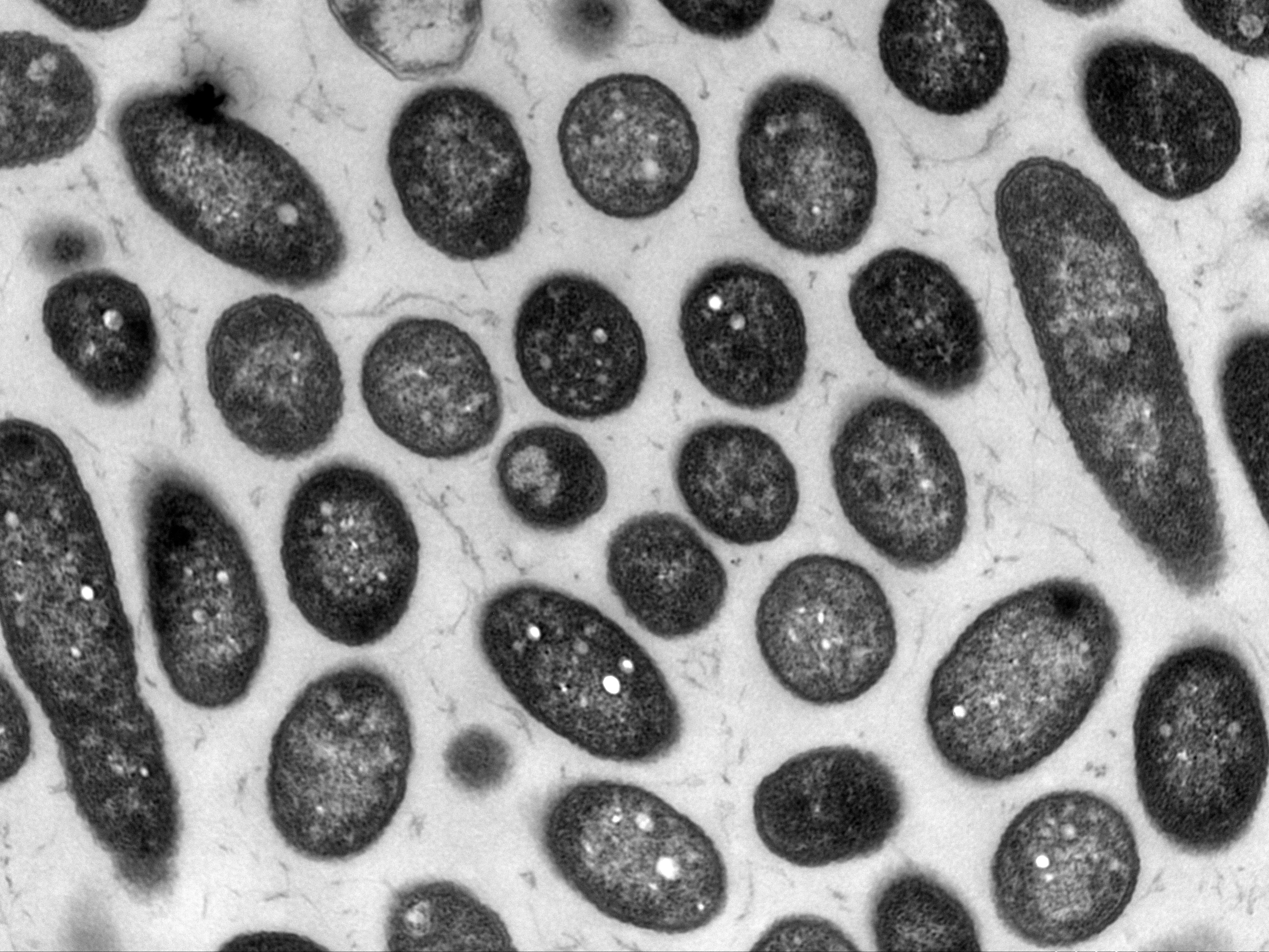

Supplement: Supplementary file 7 — Source data Fig. 5 [file 44320_2025_87_MOESM7_ESM.zip › Figure 5/5c/Suc-10k.tif]
