# Supplementary material for: Epigenetic modifications and metabolic gene mutations drive resistance evolution in response to stimulatory antibiotics
Source: Mol Syst Biol. 2025 Jan 16;21(3):294–314. doi: 10.1038/s44320-025-00087-4 (PMC11876630; doi:10.1038/s44320-025-00087-4)
Supplement: Supplementary file 9 — Expanded View Figures [file 44320_2025_87_MOESM9_ESM.pdf]

Expanded View Figures

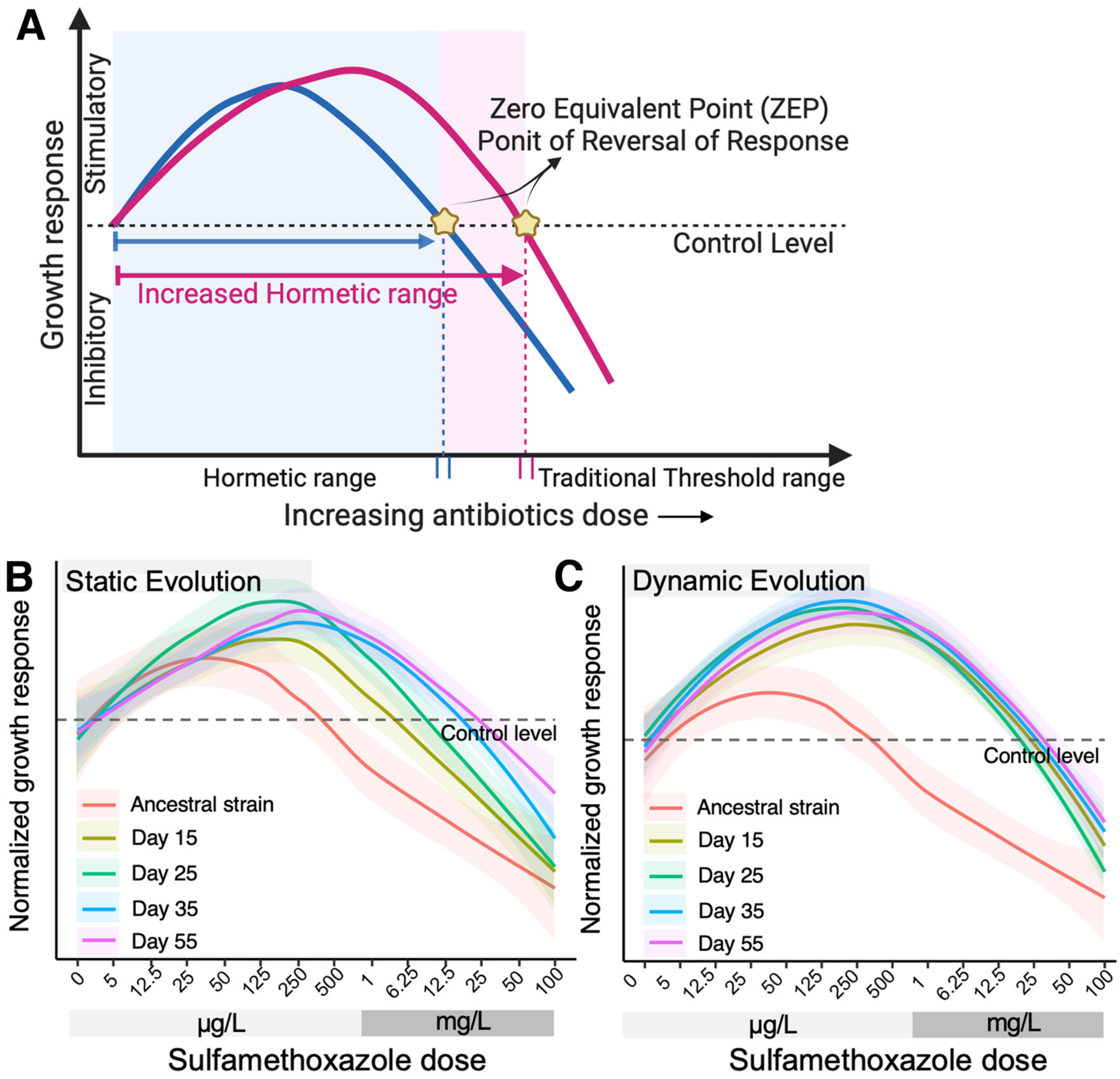

Figure EV1. Concentration-response curves (CRCs) for *Comamonas testosteroni* exposed to sulfamethoxazole (SMX).

(**A**) Schematic representation of a CRC. Fitted CRCs of *C. testosteroni* during (**B**) static evolution and (**C**) dynamic evolution at different time points: days 15 (G150), 25 (G250), 35 (G350), and 55 (G550). Growth data for the evolved isolates were normalized relative to the ancestral strain in an SMX-free environment, utilizing data from six biological replicates.

 © The Author(s)

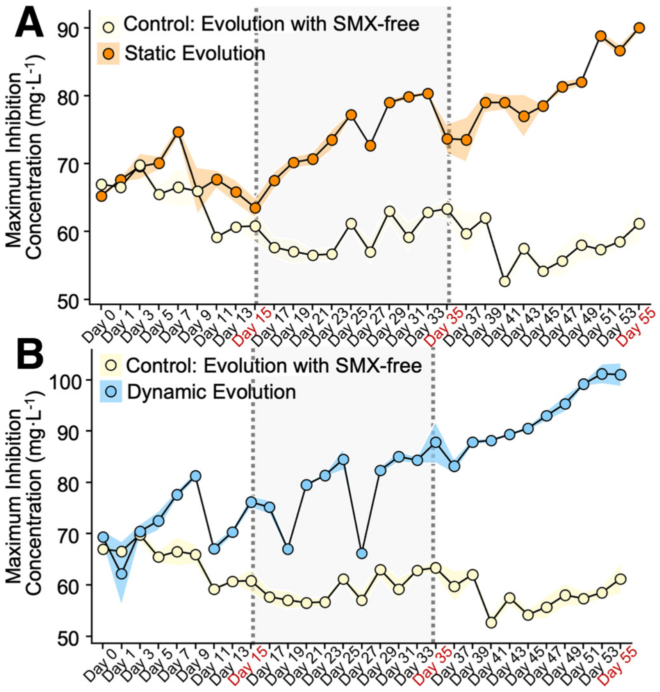

Figure EV2. Minimum Inhibitory Concentration (MIC) patterns observed over 550 generations of *Comamonas testosteroni* evolution under static and dynamic evolution protocols.

(**A**) Static evolution protocol; (**B**) Dynamic evolution protocol. Each evolutionary scheme comprised three biological replicates.

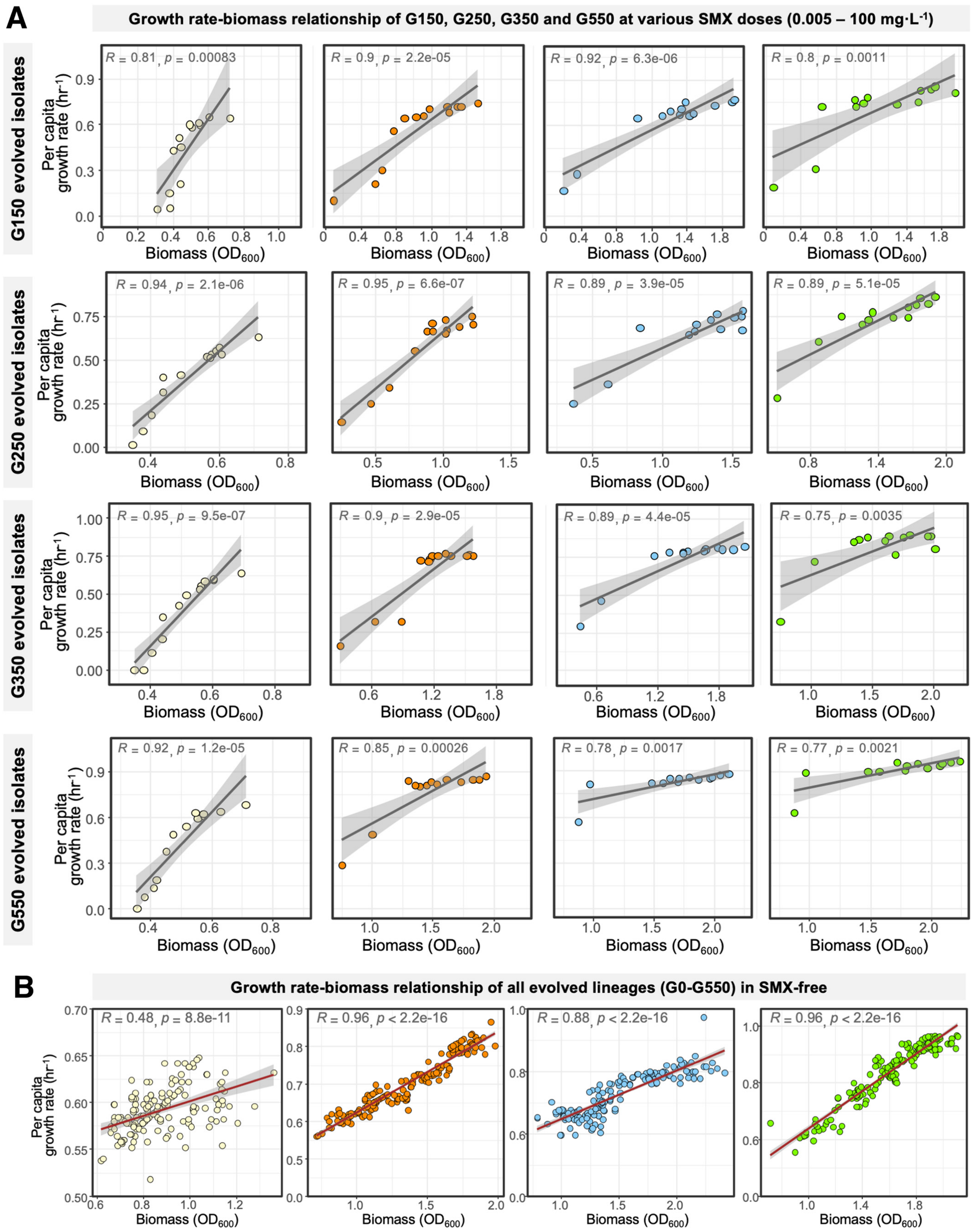
A
Growth rate-biomass relationship of G150, G250, G350 and G550 at various SMX doses (0.005 – 100 mg·L-1)
G150 evolved isolates
G250 evolved isolates
G350 evolved isolates
G550 evolved isolates
Per capita growth rate (hr-1)
Biomass (OD600)
R = 0.81, p = 0.00083
R = 0.9, p = 2.2e-05
R = 0.92, p = 6.3e-06
R = 0.8, p = 0.0011
R = 0.94, p = 2.1e-06
R = 0.95, p = 6.6e-07
R = 0.89, p = 3.9e-05
R = 0.89, p = 5.1e-05
R = 0.95, p = 9.5e-07
R = 0.9, p = 2.9e-05
R = 0.89, p = 4.4e-05
R = 0.75, p = 0.0035
R = 0.92, p = 1.2e-05
R = 0.85, p = 0.00026
R = 0.78, p = 0.0017
R = 0.77, p = 0.0021
B
Growth rate-biomass relationship of all evolved lineages (G0-G550) in SMX-free
R = 0.48, p = 8.8e-11
R = 0.96, p < 2.2e-16
R = 0.88, p < 2.2e-16
R = 0.96, p < 2.2e-16

Figure EV3. Correlation between growth rate and biomass (OD_{600}) in evolved strains.

(**A**) Across a range of sulfamethoxazole (SMX) concentrations (0.005 to 100 mg·L^{-1}) in the evolved isolates G150, G250, G350, and G550; (**B**) Across all evolved strains (G0-G550, control, static, dynamic, and metabolic evolution) in SMX-free environments. Each panel included the regression line, Pearson correlation coefficient (R), and the *P* value from the *T* test, indicating the statistical significance of the correlations.

© The Author(s)
